# Highly Conserved Non-Coding Sequences Are Associated with Vertebrate Development

**DOI:** 10.1371/journal.pbio.0030007

**Published:** 2004-11-11

**Authors:** Adam Woolfe, Martin Goodson, Debbie K Goode, Phil Snell, Gayle K McEwen, Tanya Vavouri, Sarah F Smith, Phil North, Heather Callaway, Krys Kelly, Klaudia Walter, Irina Abnizova, Walter Gilks, Yvonne J. K Edwards, Julie E Cooke, Greg Elgar

**Affiliations:** **1**Medical Research Council Rosalind Franklin Centre for Genomics ResearchHinxton, CambridgeUnited Kingdom; **2**Medical Research Council Biostatistics Unit, Institute of Public Health, Addenbrookes HospitalCambridgeUnited Kingdom

## Abstract

In addition to protein coding sequence, the human genome contains a significant amount of regulatory DNA, the identification of which is proving somewhat recalcitrant to both in silico and functional methods. An approach that has been used with some success is comparative sequence analysis, whereby equivalent genomic regions from different organisms are compared in order to identify both similarities and differences. In general, similarities in sequence between highly divergent organisms imply functional constraint. We have used a whole-genome comparison between humans and the pufferfish, *Fugu rubripes,* to identify nearly 1,400 highly conserved non-coding sequences. Given the evolutionary divergence between these species, it is likely that these sequences are found in, and furthermore are essential to, all vertebrates. Most, and possibly all, of these sequences are located in and around genes that act as developmental regulators. Some of these sequences are over 90% identical across more than 500 bases, being more highly conserved than coding sequence between these two species. Despite this, we cannot find any similar sequences in invertebrate genomes. In order to begin to functionally test this set of sequences, we have used a rapid in vivo assay system using zebrafish embryos that allows tissue-specific enhancer activity to be identified. Functional data is presented for highly conserved non-coding sequences associated with four unrelated developmental regulators (SOX21, PAX6, HLXB9, and SHH), in order to demonstrate the suitability of this screen to a wide range of genes and expression patterns. Of 25 sequence elements tested around these four genes, 23 show significant enhancer activity in one or more tissues. We have identified a set of non-coding sequences that are highly conserved throughout vertebrates. They are found in clusters across the human genome, principally around genes that are implicated in the regulation of development, including many transcription factors. These highly conserved non-coding sequences are likely to form part of the genomic circuitry that uniquely defines vertebrate development.

## Abstract 2

Highly conserved non-coding sequences in vertebrate genomes are frequently located around genes involved in development and can direct tissue-specific gene expression in functional assays.

## Introduction

Identification and characterisation of *cis*-regulatory regions within the non-coding DNA of vertebrate genomes remain a challenge for the post-genomic era. The idea that animal development is controlled by *cis-*regulatory DNA elements (such as enhancers and silencers) is well established and has been elegantly described in invertebrates such as *Drosophila* and the sea urchin [1,2,3,4]. These elements are thought to comprise clustered target sites for large numbers of transcription factors and collectively form the genomic instructions for developmental gene regulatory networks (GRNs). However, relatively little is known about GRNs in vertebrates. Any approach to elucidate such networks necessitates the discovery of all constituent *cis-*regulatory elements and their genomic locations. Unfortunately, initial *in silico* identification of such sequences is difficult, as current knowledge of their syntax or grammar is limited. By contrast, computational approaches for protein-coding exon prediction are well established, based on their characteristic sequence features, evolutionary conservation across distant species, and the availability of cDNAs and expressed sequence tags (ESTs), which greatly facilitate their annotation.

The completion of a number of vertebrate genome sequences [5,6,7,8,9], as well as the concurrent development of genomic alignment, visualisation, and analytical bioinformatics tools (for an overview see [10]), has made large genomic comparisons not only possible but an increasingly popular approach for the discovery of putative *cis*-regulatory elements. Comparing DNA sequences from different organisms provides a means of identifying common signatures that may have functional significance. Alignment algorithms optimise these comparisons so that slowly evolving regions can be anchored together and highlighted against a background of more rapidly evolving DNA that is free of any functional constraints.

One of the key decisions inherent in comparative genomics is the choice of organisms for which the comparison will be made. A number of successful pairwise and multiple-species sequence comparisons have already been carried out to identify novel enhancer elements in mammalian genomes [11,12,13,14,15,16,17,18,19,20]. Unfortunately, owing to differences in mutation rates across the genome, many slower-evolving regions show a significant degree of non-coding sequence conservation that reflects the short evolutionary distance between mammals and the slow rate of neutral divergence in vertebrates [20]. Consequently, although approximately 40% of the human and mouse genomes is alignable, only approximately 5% is estimated to be under selection, making it difficult to identify functionally relevant sequences [8]. One approach has recently been described [21] that identifies only those sequences that are identical over at least 200 bp between human and mouse genomes (termed ultra-conserved elements) and examines their distribution in the genome. Around half of the 481 elements identified showed no evidence of transcription and are therefore likely to be regulatory.

Another highly successful approach to increasing the resolving power of comparative analyses is to use multi-species alignments combining both closely related and highly divergent organisms [14,22,23,24]. By using large evolutionary distances, even the slowest-evolving neutral DNA has reached equilibrium, thereby significantly improving the signal to noise ratio in genomic alignments. Although non-coding sequences generally lack sequence conservation between highly divergent species [22], there are a number of striking examples where comparison between human and pufferfish *(Fugu rubripes)* gene regions has readily identified highly conserved non-coding sequences that have been shown to have some function in vivo [25,26,27,28,29,30,31,32,33,34]. Humans and *Fugu* last shared a common ancestor around 450 million years ago [35], predating the emergence of the majority of all extant vertebrates, implying that any non-coding sequences conserved between these two species are likely to be fundamental to vertebrate life. The *Fugu* genome has the added advantage of being highly compact, reducing intronic and intergenic distances almost 10-fold [7,36]. Without exception, all reported examples of non-coding conservation between these two species have been associated with genes that play critical roles in development. This suggests that some aspects of developmental regulation are common to all vertebrates and that whole-genome comparisons may be particularly powerful in identifying regulatory networks of this kind.

As a first step towards identifying such networks in humans, we have used comparative genomics to identify and begin to characterise non-coding sequences that are highly conserved between human and *Fugu*. A general strategy for testing whether non-coding regulatory sequences of this type are functionally relevant involves assaying their ability to up-regulate reporter gene expression in vivo. “Enhancer” assays using transgenic animals, in particular mouse, are both slow and laborious, but have provided some exciting data on the function of non-coding DNA around developmental genes (e.g., [31]). An alternative approach uses transient expression in zebrafish *(Danio rerio)* embryos [37,38,39], which are particularly suited to this form of analysis. Whilst transient expression is highly mosaic, the availability of large numbers of fertilised eggs, ease of micro-injection, and transparency of the developing embryos means that hundreds of individuals may be screened at a time. This provides a rare opportunity for increasing the throughput of this kind of functional assay.

We have adopted a medium-throughput strategy to test DNA sequences for enhancer activity in zebrafish embryos, whereby results may be cross-referenced and compared through a generalised scheme. We present functional data for 25 highly conserved non-coding sequences that are located around four unrelated developmental regulators, SOX21, PAX6, HLXB9, and SHH in order to demonstrate the suitability of this screen to a wide range of genes and expression patterns.

## Results

### Identification of Highly Conserved Non-Coding Sequences in Vertebrate Genomes

To locate conserved non-coding sequences, we masked the majority of the coding and tRNA content of the *Fugu* genome assembly [7] and compared the remaining regions using MegaBLAST [40] with the human genome sequence contained in Ensembl release v18.34.1 [41]. From this analysis we identified 19,744 sequences with similarity between the two genomes. By only including alignments of at least 100 bp in length, the number of sequences was reduced to 4,400. We then removed telomere-like sequences and transposons, and excluded any known protein-coding sequence or non-coding RNA species that may have been missed (see Materials and Methods). Sixty-five unique human sequences had matches to two independent locations in the *Fugu* genome. This is due to additional gene or genome duplications in the teleost lineage [42], where regulatory elements have been retained together with both copies of the fish gene [43]. To avoid redundancy in the human set, the longest matching sequence was retained and the duplicate removed. Finally, from the 1,373 sequences that remained, we determined whether any formed part of untranslated regions (UTRs) of mRNA molecules. Eighty sequences (approximately 6%) are situated in the 5′ or 3′ UTRs of known mRNA molecules. In addition, a similar number match one or more EST sequences, although most of these appear to be unspliced genomic contamination within EST libraries or incompletely spliced pre-mRNA. We did not remove these potentially transcribed sequences as, unlike vertebrate UTRs in general, they demonstrate a remarkable degree of conservation, and it is not clear whether they might be functional at the genomic or the transcript level. The remainder had no match against any expressed sequence in any database. This core set of 1,373 highly conserved non-coding elements (CNEs) forms the basis of this study.

The set of CNEs comprise a total of 273 kb of sequence, with a maximum length of 736 bp (average = 199 bp) and identity ranging from 74% to 98% (average = 84.3%). This is considerably higher than the level of identity seen between coding regions in these two organisms. Unsurprisingly, virtually all of the CNEs are conserved in rodent and chicken genomes, as well as a majority in the zebrafish genome. Of the 1,373 CNEs, 1,365 are conserved in the mouse, 1,316 in rat, and 1,310 in chicken, aligning to the human sequence with average identities of 97% for mouse and rat and 96% for chicken; 1,093 are also found to be conserved in the zebrafish genome, aligning with an average identity of 87.6% to the *Fugu* sequence. The zebrafish, chicken, mouse, and rat genomes are at different stages of completeness, and therefore missing sequence information may account for the missing CNEs (as well as the lower percent identity in zebrafish), although it may also reflect regulatory differences between the lineages.

**Figure 1:**
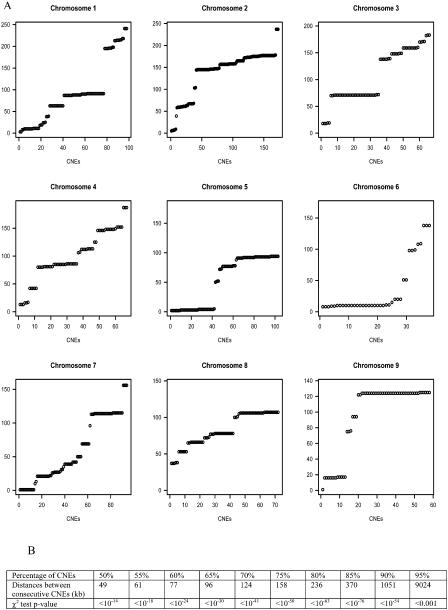
Distribution of CNEs along the Human Genome (A) Each CNE is plotted relative to its position along each of human Chromosomes 1 to 9 (data for other chromosomes not shown). The y-axis represents length along the chromosome (in megabases). (B) Distribution of the fraction of CNEs that are within certain distances of each other; e.g., 85% of the distances between CNEs are less than or equal to 370 kb. χ^2^ tests were carried out by comparing observed cluster sizes with those generated randomly for each chromosome (see Materials and Methods).

Although CNEs are found throughout the human genome in all chromosomes except 21 and Y, their distribution is not uniform; in fact, they appear highly clustered. To examine their distribution in more detail, we plotted the position of each CNE on its respective chromosome in the human genome (Figure 1A). We then calculated the percentage of CNEs that were located in close proximity to another. We found that 90% of CNEs are less than 1 Mb apart, 85% of CNEs have a neighbouring CNE within 370 kb, and 75% are located within 158 kb of another CNE. The probability that over 85% of CNEs would be within 370 kb of another in a random distribution is less than 10^−76^ (Figure 1B). By carefully examining the distribution of CNEs across the genome, we generated a total of 165 clusters, including 19 singletons (Table S1). Over 85% of the CNEs (1,172/1,373) are located in clusters containing five or more CNEs. The 20 largest clusters each contain 20 or more CNEs, comprising 43% (594/1,373) of the total number of elements.

We then looked at the type of genes that are associated with CNEs in the human genome. For each CNE, we extracted the closest gene from Ensembl and submitted the resulting set of genes to GOstat [44] in order to identify the most statistically over-represented Gene Ontology (GO) terms [45]. Critically, 12 of the most over-represented 13 terms (*p* < 0.001) relate to transcriptional regulation and development (Table S2).

**Figure 2:**
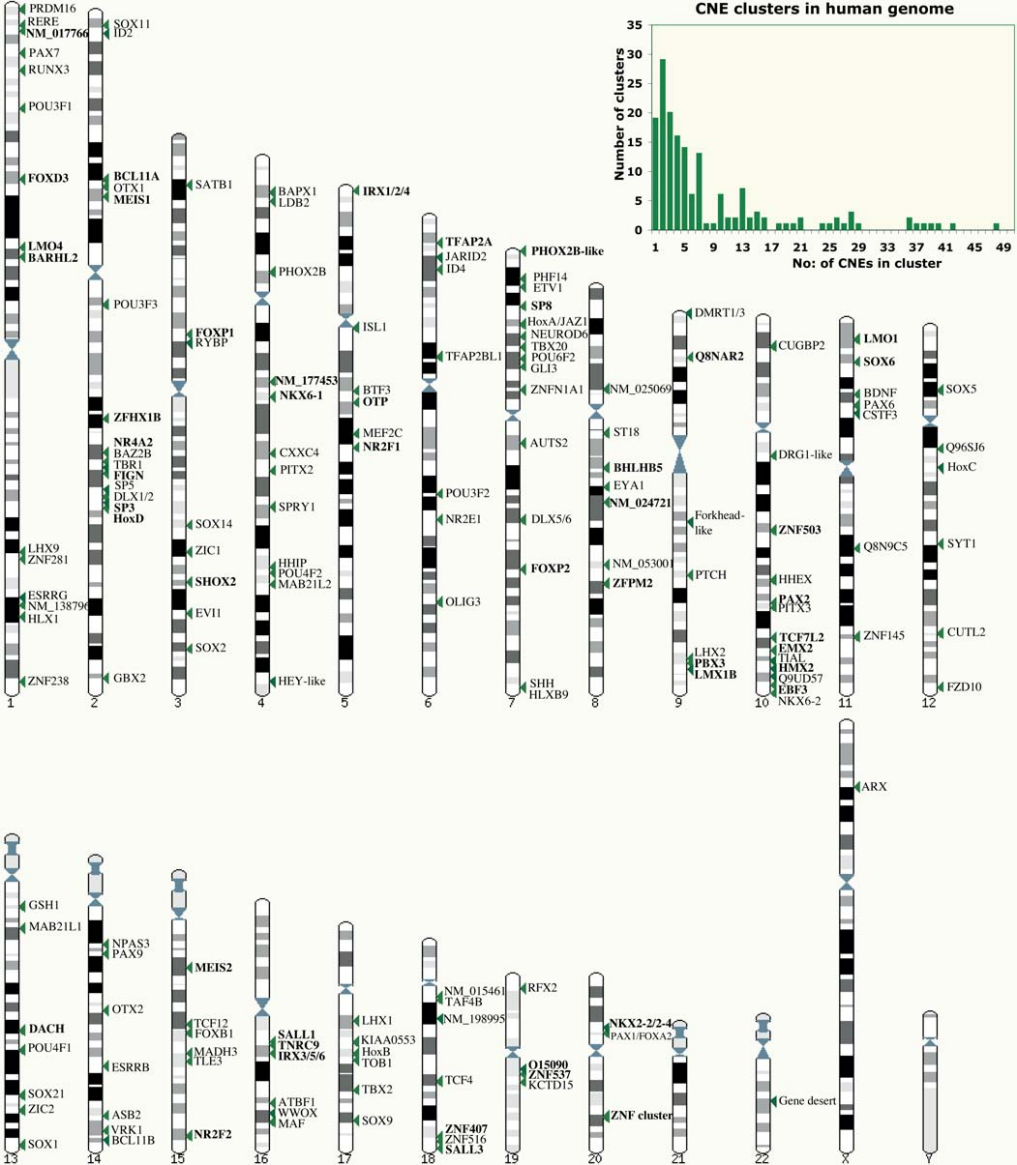
CNE Clusters Are Found Close to *Trans-Dev* Genes in the Human Genome Chromosomal locations of *trans-dev* genes that are within 500 kb of CNE clusters in the human genome (each cluster is represented by a green arrowhead). Genes in bold script are located next to clusters of ten or more CNEs. Gene names are taken from Ensembl v23.34e.1. Graph inset shows distribution of CNE cluster sizes in the human genome.

We examined each cluster in turn to see how many were situated close to genes implicated in transcriptional regulation or development (we have termed these *trans-dev* genes). Over 93% of the clusters (154/165) have a *trans-dev* gene located within 500 kb of one or more of its CNEs (Figure 2; Materials and Methods; Table S1). Of the remaining 11 clusters, five are closest to genes with zinc finger domains as identified by InterPro [46], one is in a gene desert, one maps to the AUTS2 gene region [47], and four are located adjacent to uncharacterised genes.

Whilst most of the clusters can be associated with one *trans-dev* gene, there are 15 clusters in which CNEs are located close to two or more *trans-dev* genes. In nine of these cases, the CNEs associate with a group of paralogous genes, including the HOX, IRX, Nkx2–2/2–4, and DLX clusters, although there are three instances where a pair of unrelated *trans-dev* genes are located next to a CNE cluster (SHH and HLXB9, PBX3 and LMX1B, and PAX1 and FOXA2). Finally, there are three clusters that associate with two or more zinc finger genes.

*Trans-dev* genes associated with CNE clusters tend to be located in regions of low gene density. We counted the number of genes situated within 500 kb upstream and downstream of a *trans-dev* gene, and compared this with the average for all human genes. Whereas the average for all human genes is 17, it is just six for the *trans-dev* genes. This is similar to the “gene desert” phenomenon described around the DACH gene [31]. Interestingly, the CNEs themselves are generally located large distances from their nearest gene. The average distance between a CNE and the 5′ end of the closest human gene is 182 kb (median = 120 kb), with 93 CNEs more than 500 kb, and 12 CNEs more than 1Mb, from any known gene.

A number of the *trans-dev* genes that we identified have previously been shown to have highly conserved *cis*-regulatory elements associated with them, including the Hox clusters [24,33], PAX6 [48], PAX9 [32], SOX9 [28], OTX2 [34], SHH [30], DLX genes [29], and DACH [31]. Five CNEs do not appear to cluster with any known genes in either the human or *Fugu* genomes and are located in a large gene desert on human Chromosome 22. Given that gene annotation and genomic sequencing of parts of the human genome are not yet fully complete, the discovery of CNEs here may well point to the existence in this region of an important transcriptional or developmental regulation gene with which they are associated. Indeed we find the largest number of CNEs (48) clustered around a relatively uncharacterised gene with zinc finger domains, ZNF503 on human Chromosome 10, the rat orthologue of which was recently characterised as a probable transcriptional regulator in brain development [49].

All CNEs were compared with each other to look for local similarities. Forty-three elements show significant similarity to at least one other CNE, and in each case are situated close to genes with clear paralogous relationships, e.g., the HOX and IRX clusters. The remainder of the sequences appear to be unique in the human genome.

In order to identify additional conserved sequences around specific genes for further functional assay, localised multiple-alignment comparisons were performed using the multiple LAGAN (MLAGAN) alignment tool kit [50]. This tool kit provides the opportunity to introduce genomic sequence from additional species, in this case mouse and rat, which significantly enhances the signal to noise ratio. For a random subset of 25 of the *trans-dev* genes associated with CNE clusters, stringent whole-genome alignment located 408 CNEs, whereas MLAGAN identified over twice as many conserved regions (871) of at least 100 bp in length. The whole-genome analysis was more stringent in that we used a minimum exact word match of 20 bp, whereas MLAGAN uses short inexact words to create anchors from which a more sensitive (Needleman–Wunsch) alignment is carried out. It is important to note that similar alignments on genes that are not implicated in developmental regulation do not identify conserved non-coding sequence (e.g., [22,51]).

**Figure 3:**
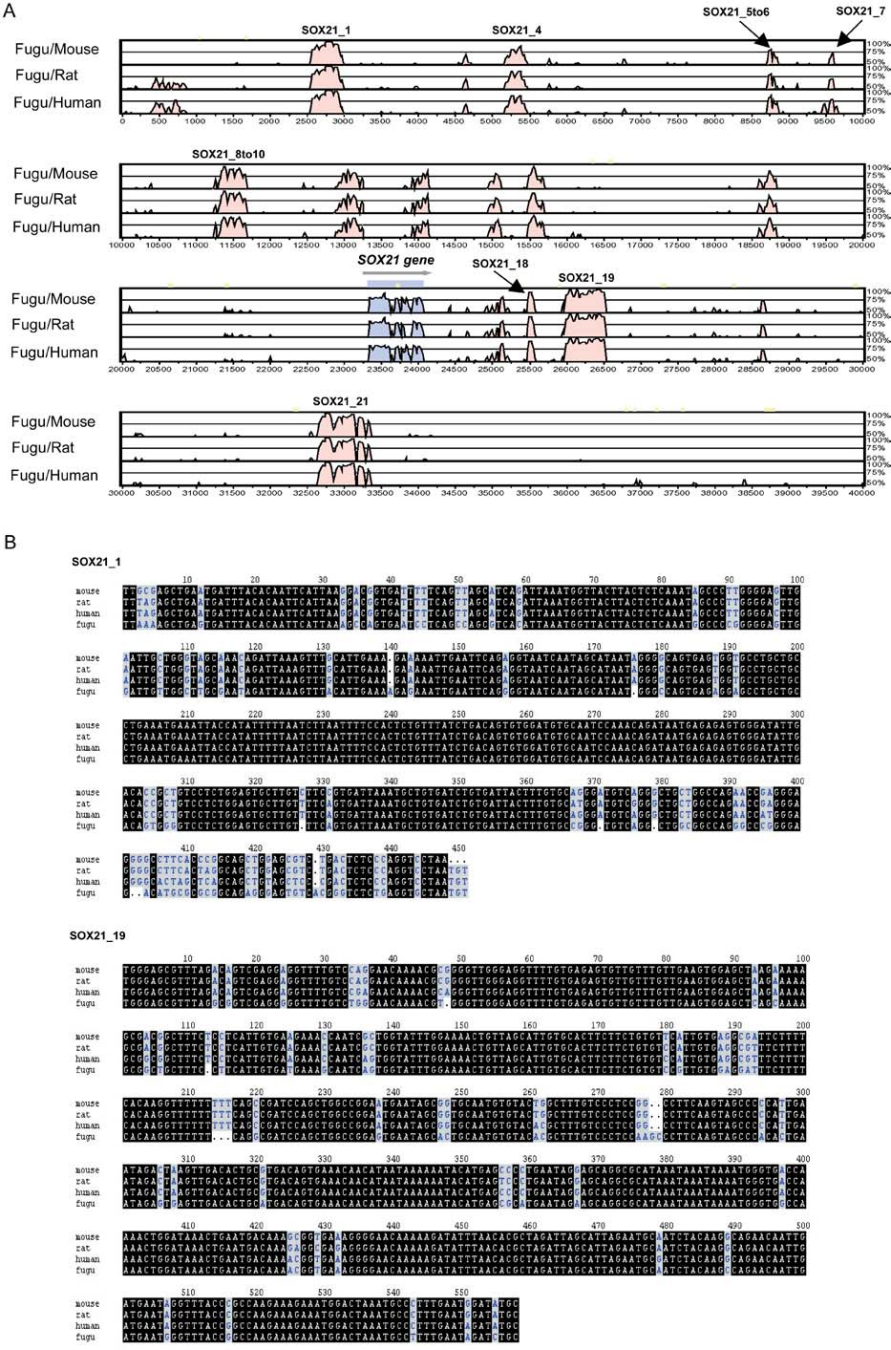
Comparative Sequence Analysis of the SOX21 Gene SOX21 genomic regions for mouse, human, and rat were extracted from Ensembl to include all flanking DNA up to the nearest neighbouring genes (ABCC4 and NM_180989 in the human genome and their orthologues in the rodent genomes). The region covering *Fugu* SOX21 (138–178 kb of *Fugu* Scaffold_293 [M000293]) was extracted from the *Fugu* Genome Server at http://fugu.rfcgr.mrc.ac.uk/fugu-bin/clonesearch. (A) MLAGAN alignment of the SOX21 gene using *Fugu* DNA as the base sequence compared with mouse, rat, and human genomic DNA. Coloured peaks represent regions of sequence conservation above 60% over at least 40 bp. The SOX21 coding region (SOX21 is a single exon gene) is annotated, and sequence identity is shaded in blue. Non-coding regions of sequence identity are shaded in pink. The eight elements that have been functionally assayed are labelled. Six of these are identified in the global analysis as seven CNEs (SOX21_8–10 covers two CNEs). SOX21_7 and SOX21_18 are rCNEs. (B) Multiple DNA sequence alignments of CNE SOX21_1 and CNE SOX21_19 between mouse, rat, human, and *Fugu*.

The alignment of a known transcription factor, SOX21, identifies a large number of conserved non-coding sequence elements in addition to the CNEs found in the whole-genome analysis. We have called these “regionally defined CNEs” (rCNEs) (Figure 3A). In mammalian genomes, the distance between the first and last element around SOX21 is over 450 kb. As is the case for a number of the larger CNEs throughout the genome, some of the CNEs around the SOX21 gene are more highly conserved than the gene's coding exon. For example, in multiple alignments of mouse, rat, human, and *Fugu* sequence, one CNE (SOX21_19) has 90% identity over 558 bp whilst another (SOX21_1) contains a 112-bp region of 100% identity (Figure 3B), demonstrating an extraordinary level of conservation for genomes separated by 900 million years of divergent evolution.

Finally we searched invertebrate sequence databases, including the whole-genome sequences of *Ciona intestinalis, Drosophila melanogaster,* and *Caenorhabitis elegans,* to see whether we could identify any of these highly conserved vertebrate sequences within the invertebrate lineage. Although many of the genes identified in our analysis have clear homologues within these genomes, we found no significant matches to any CNEs. More sensitive alignment using MLAGAN also failed to identify any conserved non-coding sequence similarity between vertebrates and non-vertebrates (including C. elegans, D. melanogaster and A. gambiae), whilst in each case the coding sequences were identified. This is surprising, given that the degree of identity between CNEs in vertebrates is higher than that of the coding regions for these genes. Thus, it is unlikely that the same set of sequences that appear to regulate important vertebrate *trans-dev* genes are found in invertebrates.

### Functional Assay

We have assayed the ability of conserved non-coding sequences identified both from the whole-genome MegaBLAST analysis (CNEs) and from regional MLAGAN alignments (rCNEs) to up-regulate green fluorescent protein (GFP) reporter expression in zebrafish embryos (see Materials and Methods). We chose four cluster regions that contain different types of developmental genes: SOX21, PAX6, HLXB9, and SHH. Elements are co-injected with a minimal promoter–GFP reporter construct into early zebrafish embryos. This co-injection strategy [37,38] is an efficient, yet simple and rapid method for identifying enhancer activity; indeed enhancer activity of elements is more striking when tested in a co-injection assay than when ligated directly to a promoter–reporter construct [37].

**Figure 4:**
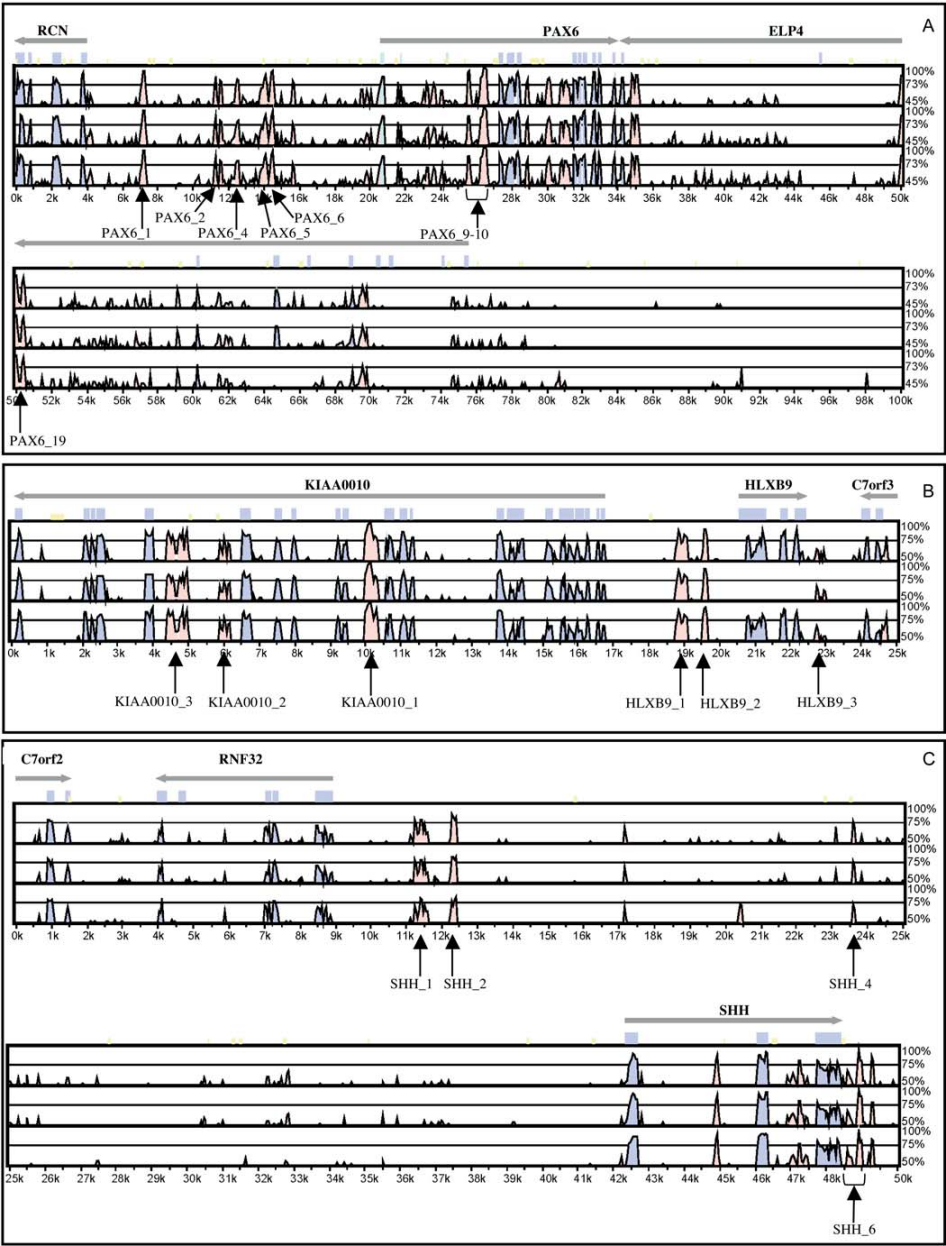
MLAGAN Alignments of Regions Encompassing the PAX6, HLXB9, and SHH Genes PAX6 (A), HLXB9 (B), and SHH (C). In each panel, human (top), mouse (middle), and rat (bottom) genomic DNA from Ensembl is aligned with *Fugu* genomic DNA from orthologous regions. Alignment parameters are the same as in Figure 2. Seventeen elements that have been functionally assayed from these regions have been labelled. The following were identified as CNEs: PAX6_6, PAX6_9–10, KIAA0010_1, and KIAA0010_3.

A total of 25 conserved non-coding regions were selected (Figures 3, 4, and S1), of which ten were CNEs and 15 were rCNEs (Table 1). GFP expression was analysed in live embryos on the second day of development and recorded both schematically and in tabular form. A mean of 188 embryos were screened for each element, compared with a mean of just over 200 embryos per control (Table 1).

**Table 1:**
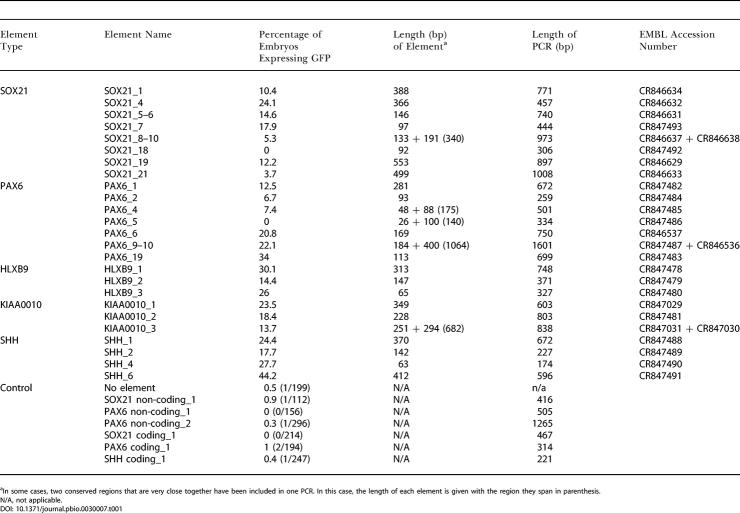
Elements Used in Functional Assay

^a^In some cases, two conserved regions that are very close together have been included in one PCR. In this case, the length of each element is given with the region they span in parenthesis

N/A, not applicable

**Figure 5:**
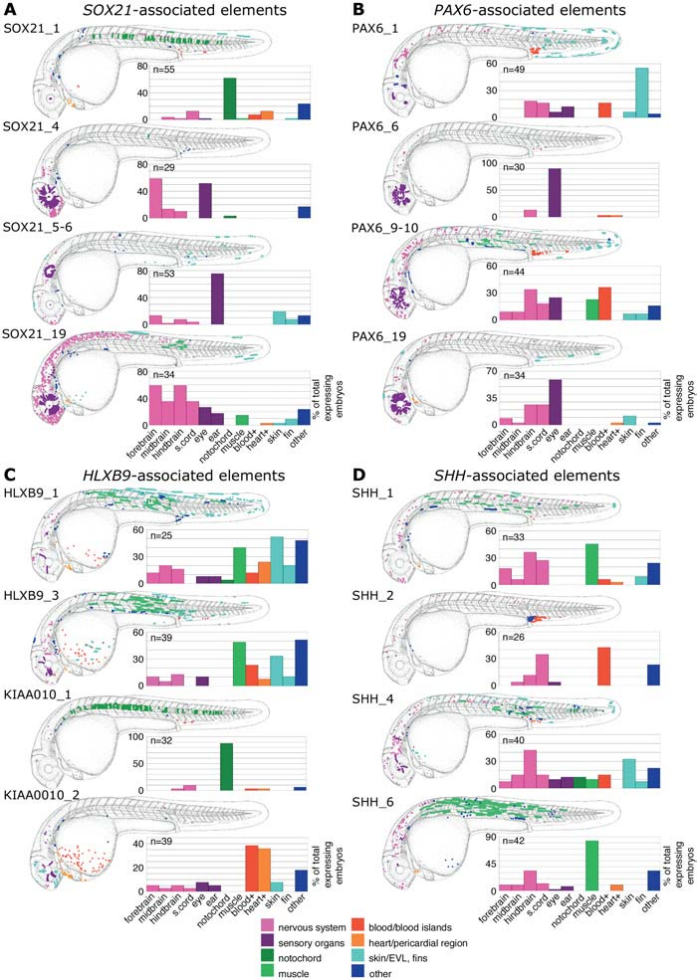
Composite Overviews of GFP Expression Patterns Induced by Different Elements Tested in the Functional Assay Cumulative GFP expression data, from *SOX21*-associated elements (A), *PAX6*-associated elements (B), *HLXB9*-associated elements (C), and *SHH*-associated elements (D). Cumulative data pooled from multiple embryos per element on day 2 of development (approximately 26–33 hpf) are displayed schematically overlayed on camera lucida drawings of a 31-hpf zebrafish embryo. Categories of cell type are colour-coded: key is at bottom of figure. Bar graphs encompass the same dataset as the schematics and use the same colour code for tissue types. Bar graphs display the percentage of GFP-expressing embryos that show expression in each tissue category for a given element. The total number of expressing embryos analysed per element is displayed in the top left corner of each graph. Legend for the bar graph columns accompanies the bottom graph in each panel; “blood+” refers to circulating blood cells plus blood island region, “heart+” refers to heart and pericardial region (Please note: Some cells categorised as heart/pericardial region may be circulating blood cells), and “skin” refers to cells of the epidermis or EVL. s. cord, spinal cord.

Controls in which no element was injected (GFP reporter construct injected alone), in which non-conserved, non-coding genomic DNA from the PAX6 or SOX21 regions was co-injected with the GFP reporter, or in which conserved, coding DNA from PAX6, SOX21, or SHH exons was co-injected with the GFP reporter produce essentially no up-regulation of GFP expression (Table 1; Figure S1). When conserved non-coding sequences were injected, up-regulation of GFP expression was observed with all but two of the elements tested, with between 4% and 44% of embryos screened being positive (Table 1). Furthermore, GFP expression was generally observed in consistent patterns, specific to the element injected (Figure 5).

In order to build up a comprehensive picture of the GFP expression pattern induced by each of the elements, the expression profiles from multiple embryos positive for a given element were overlaid onto a schematic diagram, so providing a composite overview for each element (Figure 5). This also provided a convenient format for data storage and comparison between elements.

#### SOX21-associated elements

**Figure 6:**
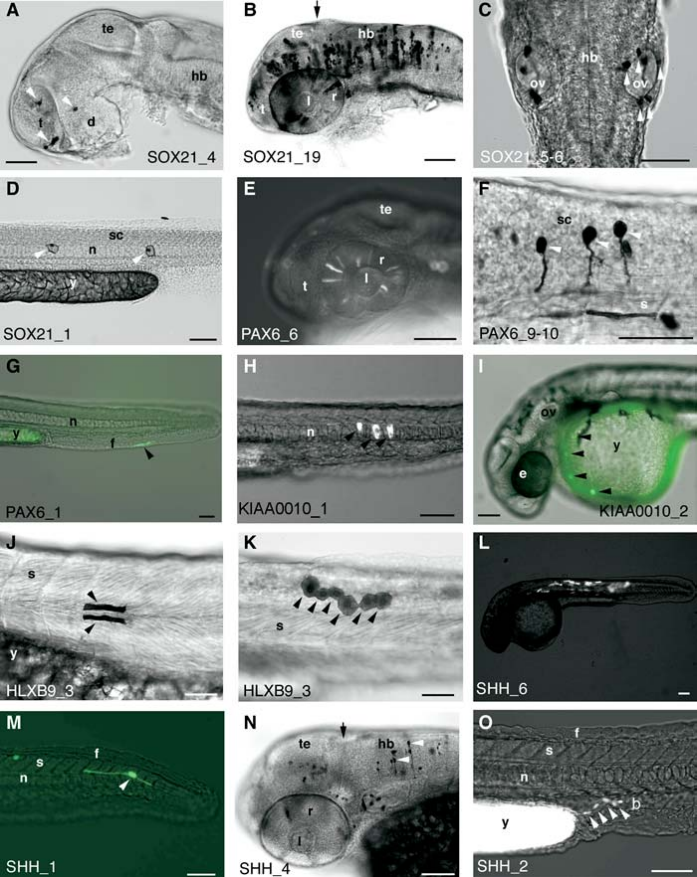
Different Elements Enhance GFP Expression in Specific Tissue and Cell Types GFP expression is shown in fixed tissue following wholemount anti-GFP immunostaining, bright-field views (A–D, F, J, K, and N), or in live embryos as GFP fluorescence, merged bright-field and fluorescent views (E, G–I, L, M, and O). Lateral views, anterior to the left, dorsal to the top (A, B, and D–O) or dorsal view, anterior to the top (C). Embryos approximately 28–33 hpf (A, D–I, L, and O), approximately 48 hpf (B, C, J, K, and N), or approximately 26 hpf (M). The identity of the element co-injected with the GFP reporter construct is shown at the bottom of each panel. Black arrows indicate the approximate position of the midbrain–hindbrain boundary; black and white arrowheads indicate GFP-expressing cells. Scale bars approximately 100 μm (A–E, G–I, and L–O) and 50 μm (F, J, and K). b, blood island; d, diencephalon; e, eye; f, fin fold; hb, hindbrain; l, lens; n, notochord; ov, otic vesicle; r, retina; s, somite; sc, spinal cord; t, telencephalon; te, tectum; y, yolk. (A) SOX21_4. Head region (eyes removed): neurons in the telencephalon and diencephalon are GFP-positive (arrowheads). (B) SOX21_19. Head region: numerous GFP-expressing neurons are visible in the forebrain, midbrain, and hindbrain. Retinal expression is also apparent. (C) SOX21_5–6. Hindbrain region: white arrowheads indicate GFP expression by several cells in the epithelium of the right developing ear (ov). GFP-expressing cells in left deveoping ear are in slightly different focal plane. (D) SOX21_1. Trunk region: two individual notochord cells express GFP (arrowheads). (E) PAX6_6. Head region of live embryo: GFP is expressed in several retinal cells. (F) PAX6_9–10. Anterior trunk region (at the level of somites 1–3): three spinal cord neurons with ventrally projecting axons express GFP (arrowheads). (G) PAX6_1. Tail region of live embryo: arrowhead indicates GFP expression in the developing median fin fold. (H) KIAA0010_1. Trunk region, three notochord cells express GFP (arrowheads). (I) KIAA0010_2. Anterior end of embryo: arrowheads point to circulating blood cells expressing GFP. (J) HLXB9_3. Trunk region: GFP-expressing muscle fibres in somite 5 (arrowheads) lie immediately dorsal and ventral to the horizontal myoseptum. (K) HLXB9_3. Trunk region (at the level of somites 13–15): arrowheads mark GFP expression in six cells forming the epidermis or EVL. (L) SHH_6. Whole live embryo: numerous GFP-expressing muscle fibres can be seen in the trunk. (M) SHH_1. Tail region of live embryo: GFP is expressed in a single bipolar neuron near the caudal end of the spinal cord (arrowhead marks cell body). (N) SHH_4. Head region (dorsolateral view): cells labelled with anti-GFP include midbrain and hindbrain neurons and cells in the retina (slightly out of focal plane). Arrowheads indicate cell bodies of hindbrain neurons, from which axons can be seen projecting ventrally. (O) SHH_2. Trunk region of live embryo: GFP-positive cells in the region of the blood islands (caudal to the urogenital opening; arrowheads) show a slightly elongated morphology, suggesting they may be blood vessel precursors rather than blood cells.

Of the eight SOX21-associated elements tested in our functional assay, seven enhance GFP expression (Table 1). Three of these enhancing elements direct reporter gene expression most prominently to the central nervous system (CNS) (SOX21_4 and SOX21_19 [Figures 5A, 6A, and 6B] and SOX21_7). SOX21_19 strongly directs remarkably widespread GFP expression throughout the brain and rostral spinal cord (88% of expressing embryos show GFP-positive cells in the CNS; Figures 5A and 6B). SOX21, a member of the SRY-related HMG-box (SOX) gene family of DNA-binding proteins, acts as a transcriptional repressor during early development [52], and is expressed in a complex, dynamic pattern in the developing vertebrate CNS [53,54,55].

Three elements strongly enhance GFP expression in the sense organs: SOX21_4 and SOX21_19 direct GFP expression to the developing eye (in 52% and 27% of expressing embryos, respectively; Figures 5A and 6B), and SOX21_5–6 strongly enhances reporter expression in the developing ear (75% of expressing embryos; Figures 5A and 6C). These observations draw parallels with prominent regions of endogenous SOX21 expression in the sense organs: i.e., the nasal epithelium, the lens and retina of the eye, and the sensory epithelia of the developing inner ear [55]. SOX21_1 strongly enhances expression in the notochord (62% of expressing embryos; Figures 5A and 6D), a domain not normally associated with SOX21 expression.

#### PAX6-associated elements

Six out of seven PAX6-associated elements tested in our functional assay enhance GFP expression (Table 1). Four of these six functional elements direct GFP expression most frequently to the developing eye (PAX6_6, 90% of expressing embryos; PAX6_19, 59% of expressing embryos [Figures 5B and 6E]; PAX6_2, 92% of expressing embryos; and PAX6_4, 100% of expressing embryos). A fifth element, PAX6_9–10, also directs reporter gene expression to the eye in a significant proportion (25%) of expressing embryos (Figure 5B) as well as to neurons most frequently in the hindbrain and spinal cord (Figures 5B and 6F).

Significantly, PAX6 is a paired-box-containing transcription factor, expressed in and playing essential roles in the developing eye; it is also expressed in the forebrain, hindbrain, and spinal cord (data from the Zebrafish Information Network; http://zfin.org). PAX6 is associated with the loss-of-function disorder aniridia. Some aniridia cases show chromosomal rearrangements downstream of an intact PAX6 gene, indicating that *cis*-acting elements can influence PAX6 gene expression in the eye at a significant distance from the coding region [56]. Indeed, PAX6 expression is known to be influenced by *cis*-acting elements in upstream, intronic, and downstream positions. For example, 5′ elements drive expression in the lens, pancreas, and parts of the neural tube [27], intronic elements drive expression in the retina, forebrain, and hindbrain [27,57], and several 3′ regions direct expression to the developing pretectum, neural retina, and olfactory region [58].

In addition to the eye and CNS, other tissues to which GFP expression is directed by our PAX6-associated elements include the blood islands (PAX6_9–10, 36% of expressing embryos; PAX6_1, 16% of expressing embryos [Figure 5B]) and the median fin fold (PAX6_1, 55% of expressing embryos; Figures 5B and 6G); these tissues have not been associated with endogenous expression of PAX6.

#### HLXB9-associated elements

We assayed six elements associated with a genomic region containing the HLXB9 and KIAA0010 genes (Table 1). Each of these elements induces GFP expression in a variety of tissues (data from four elements are shown in Figure 5C). Most notably, KIAA0010_1 directs GFP expression to the notochord in more than 87% of expressing embryos (Figures 5C and 6H), KIAA0010_2 directs expression to the blood (38% of expressing embryos; Figures 5C and 6I) and the pericardial region (36% of expressing embryos; Figure 5C), HLXB9_1 directs expression to the skin/enveloping layer (EVL; 52% of expressing embryos) and skeletal muscle (40% of expressing embryos; Figure 5C), HLXB9_3 directs expression to skeletal muscle (48% of expressing embryos; Figures 5C and 6J) and to skin/EVL (33% of expressing embryos; Figures 5C and 6K), and HLXB9_2 directs expression to the spinal cord (87% of expressing embryos).

HLXB9 is a Mnx-class homeobox gene associated with autosomal dominant caudal defects [59]. The zebrafish orthologue, *hb9,* is expressed in the notochord, hypochord, tail mesoderm, and tailbud [60], paralleling some of the domains of GFP expression induced by HLXB9/KIAA0010-associated elements.

#### SHH-associated elements

Two of the four SHH-associated elements tested in this study (Table 1) direct GFP expression most frequently to muscle cells (SHH_1, 46% of expressing embryos; SHH_6, 83% of expressing embryos [Figures 5D and 6L]). All four elements also prominently direct GFP expression to the CNS (SHH_1, 64% of expressing embryos; SHH_2, 42%; SHH_4, 57%; and SHH_6, 48% [Figures 5D, 6M, and 6N]).

The SHH signalling molecule is crucial for a number of developmental processes, and is extensively implicated in disease (reviewed in [61]). In zebrafish, *shh* and its co-orthologue *twhh* are both expressed predominantly in midline structures, i.e., floorplate and notochord. Later expression domains include the branchial arches, pectoral fin buds, and the retina [62,63]. GFP expression directed by SHH-associated elements and *shh/twhh* expression overlap in the floorplate; however, most of the other domains of GFP expression (e.g., muscle and blood islands; Figure 6O) are not reflected by endogenous expression of hedgehog genes.

## Discussion

Understanding the intricate and finely tuned process of gene regulation in vertebrate development remains a major challenge facing post-genomic research. In order to begin to understand how genomic information can coordinate regulatory processes, we have adopted an approach integrating comparative genomics and a medium-throughput functional assay. Nearly 1,400 non-coding DNA sequence elements were identified that exhibit extreme conservation throughout the vertebrate lineage. Despite a degree of overlap, less than half of the non-coding ultra-conserved regions (109 out of 256) identified from the mouse and human genomes [21] are present in this set. Most, if not all, of the CNE sequences appear to be associated with genes involved in the control of development, many of them transcription factors. A significant proportion of genes identified in this study are homologous to those identified in the sea urchin and other invertebrates as master regulators of early development, leading us to believe that they interact in GRNs. Consequently, it is extremely likely that the CNEs identified compose at least part of the genomic component of GRNs in vertebrates, acting as critical regions of regulatory control for their associated genes. Such regions would mediate up- or down-regulation of expression, effecting a cascade of downstream events.

In agreement with current GRN models, and given the function of many of the genes we have identified in our analysis, it is logical to speculate that CNEs consist of modules of binding sites for transcription factors. However, the model of CNEs as transcription factor binding sites, even for large numbers of transcription factors, does not fully explain their high sequence identity across vertebrates, given that transcription factor binding sites are generally rather short and exhibit a level of redundancy. Consequently, we have not ruled out the possibility that the CNEs may have a completely different mode of action or act in numerous different ways.

The relative positions and order of CNEs within a cluster is completely conserved in all vertebrate genomes we have analysed (generally mouse, rat, human, and *Fugu*) together with some degree of proportional compaction in the *Fugu* genome. This suggests that the CNEs might play a role in structuring the genomic architecture around *trans-dev* genes, which in turn may lead to an additional level of transcriptional control. Further evidence that genomic architecture may be important comes from the fact the *trans-dev* genes are generally located in regions of low gene density.

Alternatively, despite the lack of EST data, it is possible that CNEs are transcribed and work at the RNA level. A number of other ideas on the evolutionary mechanisms responsible for “ultra-conservation” have been suggested [21,64], involving decreased mutation rate, increased DNA repair, and multiply-overlapping transcription factor binding sites, but without more functional studies such hypotheses remain speculative. Whatever their mode of action, the striking degree of conservation displayed by this set of CNEs suggests they play critically important functional roles.

Having established a “map” of the major locations of CNEs in the genome, we were able to take a more sensitive alignment approach in a number of these regions in order to identify additional CNEs (rCNEs). The distinction between CNEs and rCNEs is purely a bioinformatics one, based on our search parameters, and we have no reason to believe that there is any functional distinction between the two sets of elements. We selected a number of elements (both CNEs and rCNEs) as candidates for functional analysis. Data from our functional assay of 25 elements from four different developmental genes demonstrate that a significant proportion can act as enhancers, inducing expression of a GFP reporter gene in a tissue-specific manner. The observed expression patterns differ among elements, but are reproducible for individual elements. Enhanced GFP expression domains frequently coincide with endogenous expression domains of the *trans-dev* gene most closely associated with a particular element, although in several instances, expression of GFP was induced in a tissue in which the most closely associated developmental gene is not normally expressed*.* This is not surprising because we are assaying elements out of context and individually. Thus, in our assay, we may have excluded another regulatory sequence in the region that under normal circumstances acts to silence the enhancer activity of an element in a specific tissue. Indeed GRN models would predict that a number of different regulatory regions must interact in order to precisely effect a particular spatiotemporal pattern of expression. One of our future directions will therefore be to assay the combinatorial effects of injecting a number of elements together. Alternatively, we may have associated a CNE with the wrong gene, particularly where there are two or more *trans-dev* genes in the same region (see below).

Whilst it is straightforward to assign CNEs unequivocally to the SOX21 and PAX6 genes based on their location in the genome, the situation is more complex for elements in the vicinity of the SHH and HLXB9 genes, which are situated in close proximity to each other in the human, rodent, and *Fugu* genomes. This is exacerbated by the fact that some CNEs may also be found within or around neighbouring genes. This phenomenon has been described for both the PAX6 [65] and PAX9 [32] genes, as well as for the SHH gene [30], where a long-range enhancer in the intron of a neighbouring, unrelated gene regulates SHH expression in developing limb buds and demonstrates the large genomic distances over which regulatory regions may act. This enhancer is identified as a CNE in our dataset and, despite its established mode of action, is located much closer to the HLXB9 gene (200 kb in human and 12 kb in *Fugu*) than to SHH (1,000 kb in human and 60 kb in *Fugu*). Furthermore, a number of elements are located directly 5′ of the HLXB9 gene, whilst others are found located further upstream, in introns of the next gene, KIAA0010. Although we strongly suspect that all these elements are associated functionally with the HLXB9 gene (e.g., KIAA0010_1 directs expression prominently to the notochord, an expression domain of the zebrafish HLXB9 orthologue), we cannot rule out the possibility that they may associate with the SHH gene (also expressed in the notochord), which lies a few genes downstream. There are a number of cases where a CNE cluster is located close to more than one *trans-dev* gene, illustrating the value of correlating endogenous expression pattern with CNE enhancer activity. However, it should be noted that in order to build GRN maps for the elements, it is desirable but not essential to know which element associates functionally with which gene.

Our confidence in the correctness of our gene assignment for the elements tested in this study is borne out by the results of our functional analysis. For the elements that we have associated with PAX6 and SOX21, there is a good correlation between tissues that express the gene endogenously and tissues induced by the associated co-injected elements to express GFP, i.e., the major sites of endogenous gene expression are highly represented in our mosaically expressing embryos (e.g., eye, hindbrain, and spinal cord for PAX6; forebrain, midbrain, hindbrain, and spinal cord for SOX21; see Figure 5). However, for elements in the vicinity of the HLXB9, KIAA0010, and SHH genes, GFP expression overlaps less often with expression domains of the associated gene to which the element has been assigned. As mentioned above, this reduced correlation with endogenous expression of their “associated” genes is probably due to the difficulty of assigning genes to elements in this region of relatively high *trans-dev* gene density.

It is likely that we have missed some developmental regulators in our whole-genome analysis owing to the stringency of our search parameters. Both the *RUNX2* [66] and *WNT1* [26] genes, for instance, share conserved non-coding sequences in humans and fish but were excluded because they failed to satisfy our stringent whole-genome search parameters. We may also have missed some elements because they were inadvertently hidden during the process used to mask coding sequence. Nevertheless, this is the first comprehensive attempt to identify the most highly conserved non-coding sequences common to all vertebrates. The use of the compact *Fugu* genome sequence, with its large evolutionary divergence from mammals, was critical in providing an exceptionally low degree of background noise in comparisons at the level of whole-genome and genomic regions.

As with any high-throughput approach, our functional screen has limitations. Since there is a negligible background level of GFP expression from our reporter construct alone, as well as from our other negative controls (see Table 1), the expression we see is most likely to be directly attributable to the enhancer properties of the CNEs. However, since GFP is a relatively stable protein [67], down-regulation of expression will not be detected during the time course of this screen; instead, expression of GFP by a particular cell indicates that expression was stimulated at some previous point in that cell's lineage. False negatives are a further limitation of the assay, e.g., tissues that develop from few cells will be under-represented and late-developing tissues or cell types (after 24 h) will be missed completely in this screen, since there is a delay between the time of onset of GFP transcription and the time when GFP fluorescence is detectable.

The proportion of screened embryos that showed GFP expression varied from around 4% (SOX21_21) to around 44% (SHH_6); this is probably due to many factors, e.g., variations in the embryonic stage at the time of injection and stochastic variations from embryo to embryo with regard to which cells the injected DNA is segregated into during cleavage. However, by combining expression data from a number of expressing embryos (an average of 30 embryos per positive element), we can gain insight into the overall pattern of reporter gene expression prescribed by each element.

In addition to seeing GFP expression in “expected” domains (with respect to the associated gene), GFP expression was also often detected in tissues in which the associated gene is not normally expressed (e.g., muscle cells for SHH_6 and notochord for SOX21_1; see Figure 5). This might be due to incorrect association of gene to element (see above); alternatively, it might reflect the importance of genomic context for function of CNEs and rCNEs. It is possible that certain regions of the genome function as silencers or suppressors, repressing the transcription-stimulating activity of enhancer elements. In our assay we are testing the autonomous enhancing function of our CNEs independent of their normal genomic context. Whilst this enables us to screen rapidly for function in an unconstrained context, it might also result in a loss of the endogenous negative constraints. It will be interesting to determine the combinatorial language of CNEs, and to uncover the importance of genomic context for their function.

Conserved non-coding sequences are likely to function as negative as well as positive regulatory elements. Indeed, it is possible for a conserved non-coding element to act as either an enhancer or repressor of transcription depending on what factors are bound to it [68]. Whether any of our CNEs can function as negative regulatory elements is an interesting question that is beyond the scope of the present study.

Zebrafish are the ideal model vertebrate for this screen. These sequences are, by definition, highly similar between mammals and fish, and the data generated are therefore relevant to any vertebrate. Given that CNE DNA can easily be generated from any vertebrate species (given its high degree of sequence identity), subtle differences between CNE sequences may be tested functionally in this system. Zebrafish embryos are both readily produced and easily visualised, allowing convenient live screening throughout development. Their transparency makes the embryos ideally suited to GFP analysis and the problems associated with mosaicism in this screen are relatively easily overcome by injecting large numbers of embryos. Technical advances, such as the use of meganuclease injection, may facilitate this further.

The combination of a comparative genomics approach together with functional screening of conserved elements produces a large and complex dataset. Efficient access, integration, and interrogation of this bioinformatics and functional data is crucial, and of increasing interest to the scientific community, to begin to characterise GRNs in vertebrates. To this end, we have submitted all CNE DNA sequences from *Fugu* to the EMBL nucleotide database and are developing a publicly available relational database in order to store, curate, and analyse data from this study as well as data generated from ongoing identification and characterisation of rCNEs surrounding *trans-dev* genes.

We have identified an important set of highly conserved non-coding vertebrate sequences that associate with developmental regulators and have provided evidence that at least some of them demonstrate regulatory function. They are likely to be implicated in genetic disease, as has already been shown for the SHH gene [30]. Their distal location from coding sequence, often megabases away, makes them strong candidates as causative agents in position effect and breakpoint disorders [69,33]. They are amongst the most highly conserved of all sequences in vertebrate genomes yet they are completely unrecognisable in invertebrates. Given their strong association with genes involved in developmental regulation, they are most likely to contain the essential heritable information for the coordination of vertebrate development.

## Materials and Methods

### 

#### Similarity searching of non-coding sequence between *Fugu* and human genomes

GENSCAN [70] (using a suboptimal exon probability cutoff of 0.1) and tRNA-scan-SE (release 1.1) [71] were used to predict coding exons and tRNA genes within the *Fugu* draft genome assembly (release 3.0; Rosalind Franklin Centre for Genomics Research Comparative Genomics Group; http://fugu.rfcgr.mrc.ac.uk/). These predicted sequences were then masked in the *Fugu* sequence by supplying them as a “repeat library” to Repeatmasker35. The masked sequence was similarity searched against human genomic sequence from the Ensembl [41] database v18.34.1 in 1-Mb sections using MegaBLAST [40] version 2.2.6 (word size 20 and mismatch penalty –2). Human and *Fugu* sequences with alignments of 100 bp or over were selected to form the initial CNE sequence dataset.

All CNEs with a significant similarity to an expressed transcript in the EMBL database or protein sequence in Swiss-Prot/TrEMBL were removed from the dataset unless located within a UTR. CNEs with significant similarity to non-coding RNAs were also removed. These were located by comparing the CNEs to the microRNA Registry [72] and the Rfam database (version 5.0) [73] using BLASTn [74]. CNEs were also searched against Rfam using the INFERNAL software. This resulted in the detection of 1 microRNA, four U1 snoRNAs, six U2 snoRNAs, three U5 snoRNAs, one U6atac RNA, three 7S RNAs, one 7Sk RNA, and one 5S RNA. The CNEs were also searched against the UTRdb (http://www.ba.itb.cnr.it/BIG/UTRScan/, which is a collection of functional sequence patterns located in 5′ or 3′ UTR sequences, but no significant hits were found. We used the program QRNA [75] to see whether any of the BLAST matches had a pattern of mutation consistent with RNA secondary structure. However, the known RNAs detected above had the most significant hits from this analysis. QRNA uses the mutational pattern in a pairwise alignment to detect non-coding RNAs, but in general the sequence identity of the CNEs is too high for this to be of use.

#### Analysis of the distribution of CNEs in the human genome

In order to test whether CNEs were randomly distributed, a new random location was allocated uniformly for each CNE within its chromosome. This process was repeated 1,000 times for each chromosome, and the average cluster sizes were calculated for the different distances given in Figure 1B. These cluster sizes were then compared to the cluster sizes of the CNEs. χ^2^ tests were carried out comparing the number of clusters containing five or fewer CNEs with the number of clusters containing six or more CNEs. The *p*-values obtained from the χ^2^ test statistics on one degree of freedom are also shown in Figure 1B. They give very strong evidence against the CNEs being randomly distributed.

#### Identification of genes associated with CNEs

The closest gene (using the transcription start site as defined in Ensembl) to the start of each CNE was determined from a list of all human genes supported by external evidence (“known” genes) downloaded using EnsMart, available from the Ensembl Web site (release 24.34e.1; http://www.ensembl.org/). The GOstat program was used to find statistically over-represented GOs in this group of genes [44], using the “goa_human” GO gene association database as a comparator. The minimum length of a considered GO path was five. The false discovery rate option was used to adjust for multiple comparisons.

#### MLAGAN alignments

More sensitive global alignment of the CNE regions surrounding 25 orthologous genes in human, *Fugu,* and other vertebrate species was carried out using the MLAGAN alignment tool kit [50]. To locate the orthologous regions in mouse and rat, local similarity searches with BLASTn were carried out using the most outlying CNE associated with each gene. The relevant genomic regions were extracted from Ensembl for human, mouse, and rat. For *Fugu* the genomic regions were extracted from the Medical Research Council Rosalind Franklin Centre for Genomics Research *Fugu* Genomics Project Web site (http://fugu.rfcgr.mrc.ac.uk/) (where there is additional mapping information for scaffolds. All sequences were orientated prior to alignment so that the coding sequence of the gene was in positive orientation in all sequences. The MLAGAN alignment was visualised using the VISTA program [76], enabling the identification of conserved sequences. Because of the larger evolutionary distance between fish and mammals, conservation was measured using a 40-bp window and a cutoff score of 60% identity. *Fugu* was always used as the baseline sequence.

#### Similarity searching of human CNEs against other vertebrate and invertebrate genomes

To look for the presence of CNEs in other available vertebrate genomes, CNEs were similarity searched against Ensembl mouse (v19.32.2), rat (v21.3.2), chicken (v22.1.1), and zebrafish (v21.3.2) genome sequences using BLASTn with default parameters. All invertebrate sequences in the EMBL database were searched in the same way using BLASTn with non-stringent parameters (mismatch penalty –1, gap open penalty 1, word size 9, and soft masking). More sensitive alignment of flanking orthologous sequence around the SOX21 gene (up to the coding sequence of the genes on either side) from Ensembl C. elegans (v21.25), D. melanogaster (v21.3.1), and Anopheles gambiae (v21.2.2) was carried out using MLAGAN as above.

#### Fish care

Zebrafish were raised and bred and embryos staged following standard protocols [77,78]; stages are described as the approximate number of hours post-fertilisation (hpf) when embryos are raised at 28.5 °C. To prevent pigment formation, some embryos were raised in 0.003% phenylthiocarbamide in embryo medium from tailbud stage.

#### Functional Assay

We assayed for enhancer activity in embryos co-injected with candidate enhancer elements or control DNA and a minimal promoter–reporter construct in a method adapted from Muller and colleagues [37] as described below:

For the preparation of DNA and micro-injection, CNEs, rCNEs, and negative controls were PCR-amplified from *Fugu* genomic DNA (see Figure S1 for PCR primer sequences; primers are represented by the first and last 20 bp of each sequence). The reporter construct consisting of EGFP (Clontech, Palo Alto, California, United States) under the control of a minimal promoter from the mouse β-globin gene, was PCR-amplified from a plasmid vector (available upon request). Amplified DNA was purified using the GFX PCR purification kit (#27–9602-01; Amersham Biosciences, Amersham, United Kingdom) or the QIAquick PCR purification kit (#28106; Qiagen, Valencia, California, United States). Element DNA or control DNA (at 150–300 ng/μl), reporter construct DNA (at 25 ng/μl), and phenol red (at 0.1%, used as a tracer) were combined and co-injected into embryos produced from natural matings between the one-cell stage and early cleavage stages, using an Eppendorf (Hamburg, Germany) FemtoJet pressure injection system. Any embryos developing abnormally were discarded before screening.

For screening of embryos and data collection, on the second day of development (approximately 26–33 hpf), injected embryos were anaesthetised in Tricaine [77] and analysed for GFP expression by observation under fluorescence illumination using an Olympus (Tokyo, Japan) IX81 motorised inverted microscope. Images were captured using an FVII CCD monochrome digital camera and analySIS image-processing software.

GFP-expressing cells were classified according to the following tissue categories: forebrain, midbrain, hindbrain, spinal cord, eye, ear, notochord, muscle, blood (circulating)/blood islands, heart/pericardial region (Please note: Some cells classified in this category may be circulating blood cells), epidermis/EVL, or fins. Cells that did not fall into one of these major expression categories (or that were not possible to unequivocally identify from morphology or localisation) were categorised as “other”. The location and tissue category of each GFP-expressing cell for each embryo was recorded schematically using Adobe Photoshop software (Adobe Systems, San Jose, California, United States), by manually drawing colour-coded schematised cells in appropriate positions onto an overlay of a camera lucida drawing of a 31-hpf embryo (from staging series by C. Kimmel, downloaded from “Zebrafish: The Living Laboratory”, courtesy of the Zebrafish CD Exchange Project; contact Mark Cooper at E-mail: mscooper@uwashington.edu;data relating to tissue category was also recorded on a spreadsheet.

GFP expression data were collected from between 25 and 55 expressing embryos per element injected. Cumulative overlaid schematised expression data for each element were compressed into a single JPEG file (displayed in Figure 5). Thus, the JPEG image for each element is designed to give an overall impression of the spatial pattern to which the element directs expression. Coupled with the accompanying graphs, the data present an overview of the spatial localisation of GFP expression as well as an idea of the number of cells per tissue in which GFP expression was detected, indicating the strength of the element's enhancing properties or the size of the cell population to which expression is directed.

#### Anti-GFP immunostaining.

Embryos were fixed in 4% paraformaldehyde and stained with rabbit polyclonal anti-GFP (#TP401 at 1/1,000 dilution; AMS Biotechnology, Abingdon Oxon, United Kingdom) using standard protocols [79] and the ABC amplification system (Vectastain; Vector Laboratories, Burlingame, California, United States). Stained embryos were cleared in glycerol, flatmounted, and observed/imaged as above.

## Supporting Information

Figure S1 DNA Sequence Data for Functionally Assayed Regions Each sequence represents the PCR product used in the functional assay. Sequence in bold type represents the position of the conserved element or elements within the PCR product. All PCR products were generated from *Fugu* DNA.
(61 KB DOC).

Table S1 Chromosomal Locations of Genes Associated with CNE Clusters in the Human Genome (from Ensembl) (40 KB XLS).

Table S2 Statistically Over-Represented GO Terms for Genes Located Closest to the CNEs (67 KB DOC).

### Accession Numbers

All 1,373 CNEs (CR846105 to CR847477) and 16 rCNEs (CR847478 to CR847493) have been submitted to the EMBL database.

## Abbreviations

CNE: conserved non-coding element
CNS: central nervous system
EST: expressed sequence tag
EVL: enveloping layer
GFP: green fluorescent protein
GO: Gene Ontology
GRN: gene regulatory network
hpf: hours post-fertilisation
MLAGAN: multiple LAGAN
rCNE: regionally defined conserved non-coding element
UTR: untranslated region
