# Supplementary material for: Highly Conserved Non-Coding Sequences Are Associated with Vertebrate Development
Source: PLoS Biol. 2004 Nov 11;3(1):e7. doi: 10.1371/journal.pbio.0030007 (PMC526512; doi:10.1371/journal.pbio.0030007)
Supplement: Figure S1 — Each sequence represents the PCR product used in the functional assay. Sequence in bold type represents the position of the conserved element or elements within the PCR product. All PCR products were generated from Fugu DNA. (61 KB DOC). [file pbio.0030007.sg001.doc]

SOX21_1

AGCCGTCAGTAAACACTTAGCTCCCTTTAAAGGGCAGTATTTAACCAGGCCCATATTTGGGCGGTGAACTGCAGTGATGTCCCTTTTGGAATTACATTCTGAAAGACCCCATCAGACC**CTTTAAAAGCTGAGTGATTTACACAATTCATTAAAGCCAGTGAATCCTCAGCCAGCGTCACATTAAATGGTTACTTACTCTCAAATGGCCCCGGGGGAGTTGGATTGTTGGCTTGCGAATAGATTAAAGTTTACATTGAAAAGAGAAATTGAATTCAGGGGTAATCAATAGCATAATGGGCCAGTGAGAGGAGCCTGCTGCCTGAAATGAAATTACCATATTTTTAATCTTAATTTTCCACTCTGTTTATCTGACAGTGTGGATGTGCAATCCAAACAGATAATGAGAGAGTGGGATATTGACAGTGGGGTCCTCTGGAGTGCTTGTTTCAGTGATTAAATGCTGTGATCTGTGATTACTTTGTGCCGGGTGTCAGGCTGGCGGCCAG**GGCCCGGGGAGACATGCGCGCGGCAGAGGGAGTGTCACGGGTCTCTGAGGTGCTAATGTACCTGACACAGCACTGACAGCCCTGGAAACCCTATCAGGGACCGGCGCCACACGGCAGCCGCCACAATACCCAGCCTCCCGGCCGTATCTGCATCAGAGTCAGCACATATCCTCTGCTCCCGCCGCCGATAACAGCCGGGCAAGAATGGCCGCAACAAAGTTTGCACAATAAGTATTATTAGGGCCGGTACCTTCCTGCGGCGAAC

SOX21_4

CTGGGCCATGAGAGCGGTCGATGTTCTGTGCATGTAAACGTGCAGCTTTTGGGTAAAGGTGGGGACAGCCTGTGTCCCCCCAT**TATTGTCTGCTCGCATTTACGTGGGTCCAAGGGCCCACTGTGTCTTTTCGGGTTACTTTCTCATCATTTCTCCTAATGTAGAGACCCACAGGGTTGGTGGCAAGCGCACAATGCGCCTGTTGTATGGTTACTGTCAGCTCTCTCAAATAAGGCTTCCTGTTCATTAGACTTCTTTTCACCTCTGCACAATGCGCCGGCTTTGCAGCAATAGTGAGTTGACGCCCATCTCTTCAATGCCATGTGAATAGTTTTAAATAGTGTTTATGCAAGCCATTGTCGTCCATTGTGCGTCCCGTCCTAATGCACAGTGATTTGCCTGCTTTTGTAGCCTTGGGATACATTATCTTTCCTATGTTGCTTCAGATG**CATGCCTG

SOX21_5-6

CCACGGTGACCTTCATGCTGTTCTGACTTGTTATCGCCGTTAGCTTGGCGCAGATTGATCCATAGATTGCGAGAAGAAAGGCCACATATTGCGACGGGCCTCTTTAATTGTAGACAGGTGTAACTGGATTTGTGCAAGGCCATTGAAAACACTGTGTGCTGTAGTGTGTCGCCCGGGAAAATAAAGTAAGCCTATTCAAGTATGTTTCTGCCATTTCATCAATAAAGGATGTTGGTGTCATTTTGCTGCTGCTGTGGGTTGAAATATTCAAATCCAGCCTGACATCTGAGACTACACGTCACCAAAATATCTCTTCTATTTATTTATAAAGGGGGAAAA**AAGGCACTGACCATTGGGGCATAGCCAATATGGAGGTTAATCCATTCTTGTCTATACTGGGATGCTTATGGAAGCATATTTGCTTAGGGCAGCCATGCAAATTATTACTTACTGCCCTGTAAGCTGATAACACAAATTAGATACCC**ATTAAAGTTGTACAATGTATATAGAATGAGAGGAGATTGCAAGTGATACCTGAAGGGGTGTTTAAGTCGCATGGCGCCGAAATACGCCTCGTGCTGGCAAACTTCAAAAGTGGAGAGATGTTTATCCTGGGGACGAAAGATGCATAATTGTAATCTCATATGCTCAGAAGCAGCTGGCAGGTGGAGGGAAGCACTGCGCATGTTTATGAATAGGAGTCATTAAAGGGACTGGGGTGCATACCTGCACGGACCGTT

SOX21_7

CGGGTCACAAATCGCCAAGTAAAAGCAACACAGCGTGGTTGAAATACACTCGCCTCCATCCCTTTCTCCCCCCTTCCCTTCTTCCTCCTCTCTTTATAT**TCATTAGATTCATTATAGGCCACAAAGGATTGCCAACAAAACAGCTTGTGATTTTAGTTGAATTCATCCCCCTCTGAGGCCTGCAGGGAAATTAAAG**TAGGTGACTATCACTGGAGGGGGAGCTGGCTTGACACATCTGAGGCTTTCAGTGATGTTAATAGTCAAGCGTATCCAAAATACACAGGAGCTGACCTCAAACAGATTGTGGGGGTTTAAAATCATTAGTTTGCAAATGAAGCAAAAGTACATTTGGTTGTATGGTAGAATCAGCCCACTCTGAACCTTTGATTAGTGTTTGTCAACTCCCCTTCCTCTTCATACTCCCCCGCCCACTCCCCAATCCAG

SOX21_8-10

GCAACATCTCCCTCTGCACATTGATATTTTTCATGAAAGGCCCTGAAATTGTACAGAACCATTTCCACGAGTCAAAGGAATCAATTCAGAGGAAAGTTGAAATGCTATTGTAAAATGGCCTTTTCAACACTCCTGCTCCAAGATAAACAATTTCCCATGAATATATGCCCCCCCGCCGCCCATCTATCACCTAAATCAGACTGTGTCCCGCACCGAGCCCATTGTAGTCTATTAAAGGAAATCCTTCCCTGTGGCAACCATTTTGCGGGTTTCAAGTAGATAAATGCCCTCCAAGAGGGATAAGAATGCATTTCGACAAAACTGCATTAAAGAGTAATGTTGGTCAACCGCTCTGATAAAAACTTCAGAGTCACAATTCTCTTTACAGCCGGCAAAATGCATCGACACTGCGTAAGGCTGATGGGTCTGCTGG**GGCTGCCTACAGGTTAGCAGGCCTTATTTCCAATCTCTGACAGCAATATGTGTCGCCGCGGCTTCATCCCCAACACGCGTGTTCAACACAAGCCGTGGTCACTCTATATATGGAAATCTCTGTCTTCAGGGCG**GAAAAAAATTAAAAAA**GGTGAGATTGGCACTTTAACCCCCGTTCCCCCCTGAAAATACGTTTTAGTCCGATACATTATACTACTGCGAATTATTTATCAAGGAGATACAAGCTTGATATTTCAGGAGGGATAATGAGTTGGTGAGGTGGCAGCTGGAATGTTCCGGTCCAGCGAGCAGCATCTATGAATCGAGGGAAATGAAGTAAA**AATGGCTGGCTGGGACGCTCCATGTGCAGAATGTGAAAGAGTGGAGTGCAGTGTTTAGGAGGTGGTGGGGGGGGGACTTTGAATCTCTCTTTTGTGTCCATCACGGTGCAATCAAAAGTATCCCTGCGAGGCAGCTTTGGTTTTTGACTGTAATTGTTTTTGCACAGGTTTGAAGGGGTTTGAGTGCAGATGCGTGAGAT

SOX21_18

CACGTGGACGTGAGAGTGCACAAATTCTTTTCACATTAGATCTGTGACGTGTGCTGCCTGCCGTTTCTGCCCCCTTTCTTGGGTTACACTGAA**AAATTAATTCATAAGGGTCTGAGCGATTATGCAAAACTAATTTGGACAGTCCAGGGATAATTATCTCCGTCACGGTTAATTAAATCCTTTCA**CCGCGATGTTCCCCATTGTTACACTCTTCTTCTCGGCAGAACGTCTTTCATGGCTCGAAAATAACGCGCAAGTGACCTTGTGTTTCTGAAGGCGAAGAAAAAACGCGCAGATCTGTCAGGA

SOX21_19

CAGTAAAAGGCTCGATCCTATATTTGAGAAGAAATGGCTCTGAGCTCTCGAGTTTGAGCATTTTATGCTTTCTTTTAGCTAATCATGTTTAAAGCTGCAAATCTAAATTCTTTTAAAAATACCAACTTTTTATTGTTTTTTTTTTATGATTTACATTTTAAAAAGCATGCATTATGAGTCCAGTTTTCACGTTTTTTCCCTTTCTTTGGGCTTTATTTTTAAAATTATATTTTCCTTTCTTGGGGTAAAAAAGTGGGAAGAAAGCTGAAGGAACTTTTTTTTCCCCCCTCCAAACTC**TGGGAGCGTTTAGGCGGTCGAGGGGGTTTTGTCTGGGAACAAAACGTGGGTTGGGAGGTTTTGTGAGAGTGTTGTTTGTTGAAGTGGAGCTCAGCAAAAGCGGCTGCTTTCCTTCATTGTGATGAAAGCAATCAGTGGTATTTGGAAAACTGTTAGCATTGTGCACTTCTTCTGTGTCCGTTGTGGAGGATTTCTTTTCACAAGGTTTTTTCAGGCGATCCAGCTGGCCGGAGTGAATAGCACTGCAATGTGTACACGCTTTGTCCCTCCAAGCCCTTCAAGTAGCCCACACTGAATAGAGTGAGTTGACACTGCATGACAGTGAAACAACATAATAAAAAATACATGAGCGCATGAATAGAAGCAGGCGCATAAATAAATAAAATGGGTGGCCAAAACTGGATAAACTGAATGACAAAACGGTGAAAGGGGAACAAAAAGATATTTAACACGCTAGATTAGCATTAGAATGCAATCTACAAGCCAGAACAATTGATGAATGGGTTTACCGGCCAAGAAAGAAATGGACTAAATGCCTTTTGAATAGATCTGC**CTGTGTTTTTTCTGTCTGAGATGGGGAGTGAATGCAAAAGGTGG

SOX21_21

GAGGACCCACGGCCAGTGTGGTTGCATTTTTAACTCAGAGACTCTTAGAGAATCGCATGTTGGAGAAGGCAGAATGGCAGGAGAAGGCACATTTTCAGAGTAAAATAATAGATAATTGTACTCCAACAAAAAGGAGGAAAGTATGGGCAAGAGAACTGAAGAGCAGGAGGAGATGGGGAAGGAAAGGGCTCTGAAAAGAATAAGAAGAATTCGGTGCTGCTTCTTGCAAGGCAACAGAAAAAAAAACATTGATGACACCCGGTTCCATTCTTCCG**CTCTCCCCTTTCTCTGCTCCTCGCCTCGCAGCACGGCGGGCTAAGCTGGGAGCTGTAATGTCATTAAATTGCAAATGCTTTTTTTTCATTTCCGACACACACTGTTTACTTATCTGGCAAAAACATATGTTGGAAGGAATCCGTTAAATTCTGCCCATTGCCTTGGGTGAAAGTGCAATAGAAACGGGCCCACTGAGCTCCTTCTGTCGTCTCCTTTTAGCACTTTGCCTTATCTACAAGGCTGCTTAGCAGCAAACTGGCGAGTTCAATGAAAAATGAAGATACAGTGCAAGATTAGGGAAGAACAGTGAGGGGAGAAATGCTTGGAGAGAGGGTCATCCCATTGCAAATGATTTCACTCCTCTCCATCTGCATAAATGACTATTTTTAAGGCATCTCCAGCGTCTGTATATTTTTCTCACTCTGCCACACATTTAATTTCACGCCCCGTGTTCTGAATATGCTTAAATGCATATCTCTGATGCACCTTTAAGCCAGA**GGGAAAGAGGATGGGGGGGGGAGACTGGGGAGGGTACGAAAGCAAAATTAAAGATAAAACGGTGTGATTTGCACTACAAGTCTATTACTTTATGGCATTTACATATAGTTTACCTCTGCAATTCCAAGATTTTTTTTTCTAGCACCTTCCTATCAGTGTTTGGGCATGTATAAATATGTATAAATCTTTGTTGAGAAAAATAGAATTCCTAGCACCGCTCTGGAAAGTTTGACG

# PAX6_1

CCCCGCACTTGCTCTGAGATCACAGCGTTGATCATTTTTACCTGGAGGTAAAGCAGAGGATTTCATGAGCGAGCCTTCTCCCTCCACAGGCCCTCCGATTCTGCACAGATTAATAAACTTCTCATTGGTGCCACATGGGAGAAAAGAAACCTTTGCTCTGTTTAATGACAACTGTAAAGTGGAGCCAATCATGGGATGCCTCCCGTGACATTTCCACGGCGCTGTCCGCTCCACTTATCTTAG**ACAAATGGCCGTGCCAGGCCGCCATCGAGATGAAGCGTTTTCCCCGAGTCATTACGTCTGTCACCGGAGCCATTCCCTTACTGAAAAATCGGAAACAATAAAGGACGACTGAGCCCTTAATAACTGTGAGGGAACATATTTGGAAAATTGCATTGAAGCAATAACCTTTTCTCCCTGTAATTAGATGACAGAGGGGTCCTGGTTTTTAATTAAGTCAACAATTTATGGTCTGGCAGCCATACCTCAGCATACTGATTAGTGTAGAAGGGATCAGGGAGCA**GCGTCGCCTCCATTTGAACACATTTGCTCCAGTAATTAGGGTGGAATATTGCAGAGGCAGAGAGCTCCGCGTGAGCCAGGACGTTTTCAGGACTGCAAAGATGCGTTTGTCTGGAGGCTCATCAAGAAACAACAACATGGCAGTAGACC

### PAX6_2

CCAAACCCCACATTTCAAATCCCTTGTCTGGGTGT**TTGTTTTCATATTTGTTTGACGTGTATTGATTTTAAGGCATCTCTGGGCTTTGTGTCGCTGGGGAGAGAAAGCCCCCGGTCACCCTGCATTAG**TGTGATGCCTGCGCACTCTAGCAGGACATCTGATGACCC

PAX6_4

TCTCACATTTGAAAATGTTTGGATCTGACCTCCCCACCCAAGTCTTATTTCCTAAAGGAGAAAGAAAACACCAAAACAGCAAATCCGTAGAAATATACATTCCGGAGAATTACATGTTGGCCATTTAGAACGCGCCACATTATGCCACAATATATTGTGCCAATGGTCTGAAAGCCATTATGAGGGCGCCGCTTTTCTATTCAGACAGAGAAACTGCAAATAAC**AATCAGTTCAATTATCCTCAAAATATTTACTGCAGGAGCGGATTTGTCATGCGGACGCAGAGAAGGAGCGAAGCGACGACAAGTGATTTCTTGCTGCACTGTGCGCGTCCATTTTCCAGACGGTTTGTCGTCGTTATTGCCTGATTTAGTGTGTGTTGTTTTACACGGCCCAGTC**ATCAGTGTGGGTCCACGTGCAGCGGTTGTGCCGCTGCATCCATTTATCCGTATCACCCAGATCTGCGTGAGTTTGTCACATTGCTTGTTTTATATTCTGTCG

#### PAX6_5

CGCGCGCTGATTGGACTCACACCGATGCAACCCTGTGGTTCTGCGCAGATCCAGCCCAGCATCCTTTAGCGACGCGCACACCTCTCCGCGCGTAATTGACTGCGTCGTGGCTGGATCCCCCGTGCGCGCAACACTTTATTTTGAACGGTGCAATACGTTTCCATTAAACGTTATTAAAGCGCGCGGA**GTGACGAGCTCTCATTTGTCTTGTCAGGACATCTGACAACCTCGTTAGCTCCCATTTTCCCCTGTCAGCTCACAGCGCGACACGGACACTGACTTCAGCCACTCCTGGCAAGCGAGTGATAAACGCGCGCACTCAGCCCT**GCGCAAA

# PAX6_6

CCTCACCACAGCACTCCGAAGGTATCGCAAATTCGGCCATTATGGAAATTTTCCTTATTTTATGAAGAAAGGGGGAAATAATCGAGGGAGAAGAACA**CTTAATGGCATGGGATCCTTCCGCTGACTGCACATTCAGATGCAATTGTAGATCGAAGTCAGACTTGTCACGTTGAGCCAAAGTGAATTCCTAACATCCAGGACGTGCCTGTCTACTCCGGACAAATTGCATCCAATCACCCCGAGGGAATTCGGCTAATGTCTCGATC**CAGGGCCGGGGAGCATGGAGAGAAAACAAATCAAACTCTGCACACAGAGCGTGTAGGGCTTATTAAAACATTTCACACAGGGGGAAAGAGAGCGCGAGAGAGGGGGAGAGCGTGTTATATGTGCTTGTGTGTGAGTGTGCGTGTGTGTGCGTGTGTGTGTGTGTGTGTTTGTGTGTGTTCGAGAGAGGGAGGAGGTGTAATTATATTATCTTTGGACACTACGTCTTAAATTGAAAATATCAGATGAGCAATCCAAACATCTCTGCTCGCCACGCGCCTGCTGTGTGAGCAGCCCAGCAGGCCCGAGCATCGTATCCACTCTGGCGGCATCAGCTCCCAACTTTGGAAAACACCTTTTTATAGCCACTTAACGTTAAGACACAGTTTGGCGTGTTTCGACGCACGATTCCACATCCAAGCCGGAGCTATTTTAGAGAGACCAAAAGATTCAGAATTCAGGATTAACCGTGAAACTTACCTTTTC

#### PAX6_9-10

TGACGTGTATGTGCTGTAATCTGTGCGCCTTTAACCTCAAACAAAAACAAACACGATAAAATAATCTGGGAATGGGATCATTTCGGATCAAATATTGGGAATTGTAGCCTCTGTTGCGCTGCGCAGTAATGAGGCCTCAGCAGAACTGCGCGCTCCCCGGTCGGTGATGATGAAGATGATGATGGTGATGATGGTGATGATGATGAGGGCGCCGATGCTCTCCTGGCCTTGGCGTGTGATGTGTGGTGCGAATTGTCGAGGAGATATCATTGGGAGCGGCCCTTTGGGTTGTCTAAAGGTCGCGGCGGCCTCCGCGGCTTTTGTGTTAATAACTCCTGGCTCTATAT**GGTGGGCAGCGTCCAAATTGACACCATATGTTTTCCTATTAGTCGCCTCGCAATAGAATACAAGGCGCATAATCAGCGCCAGCGGGCGAGATCGACTCTGCTCACTTGAGGGAATTGTGCCATGATTAAGACTTAACCTTCGGAGAGTTGGGTTTTTCTGTCCTTATCTGACGGCTGTATCGAT**TTATGTGATAGTTGTAAATCAATCAGACGTGACGGAGAGGCTGCAGAGCGGCGCTGCTAATGCGCAGGGGCCAATTTCAGCGTAAGAAGTCTATTAATTCGGCTTAATCCGGGAATTACCCAAAGAGCAGGAGGGATGATAATGGGTTTACTATCAATCAGATCCTCGCAGGAAGATTATTACACATTGCGGGAAAAAGAGATGTGTGTTAAGCAGGTTTATTTTCGCCGCGCACAGGCCGTTTTTGACCGTTTATTGGGAATAAAATGTGCGCGTTCAGGGAAGCTCCTGTGACATATTTCATAGCAGAGACGCCCGCCGACTGCACACAACAGGGCTGCAGTGGCTGCGAGCATTTGTTGTCAAATACAAACAAGGGGTGGGTGGGCAGGAAAGAGGGGGGAGAACTACTTGTCACTTTTAATATGTTAAGAATCCAGGCGCGCCCTCGTTTGTGTGGCCGCGCGTAGCCCCTGGCCA**CCTTAAAACGAAGTCTCCCTCCGAGGGTAAACGAGTAGCGTCCAATTTTGTCTGAGTGATATCCAAATGCAGACAGAAAGGGTCGTTTTATCATGCTACTATTTGTCGTGACGATGCGATTTTCAAAAGCAGAGCGGTGTCATAAAGTGACATGCCTGCCACAAGTGCTCCAACTGATCTTTTCAATTAGCCTCCCATGCATGATCCGAGGCGACTTCCGCCTATTTCCAGAAATTAAGCTCAAACTTGACGTGCAGCTAGCTTTATTTTAAAGACAAATGTCAGGCAGGCTCATCATATTTTCCCCCTCTTCTATATTTGGAGCTTATTTATTGCTAAGGAGCCTCTGCTCCCGGAGTCAATGTACCGGGCGGCAGCGCAGGGGAAGGCAGCGGAGAGG**CAGAGCGCACAGGCACTCCGGGGAGTCGGCTCTCCTGTTTGGCTTCAGGTTTGCGGGCTTCTCTTCTGCTTTAGCGGCATCTGGCGCAACGATGATTTGAAAGAAAAGGCACTCTTTAAAAGACATCAGCACAATAACAGGTATTTCACAGCCGATGCCTCCGCGGTGCCTCGTCTTCGTGACGCGTAAC

# PAX6_19

AAGATCACAAGGCAAATGAAAACATTTTTCATAGTTTAAATTCATAGAAATTTTGCTTCAGGGGTTGCCATGGCGACCCCTCCTCACCTGTGCTCACTATTGCTGACCAACGTATACCTATATATATATATATATATATATATATATATATATATATACCTGTATATAACAAACATTGGAATGCAATTAAAATAAAAAGCATTTGCCTTGGTGACGTGAATCTGAAGGTAATTGTGTGTCATGACTGGACGATATTTGCAGTAACAAGGCAAAAACAATGGCTTCTCATTCTATGGGCAATACATGCTGGCAAACACACTAACTTCACTTGCCAAATCCCCAAATTAACACCTTTTTGGTGAAAAAGCTCAATTTGGAGCACATCACACCAACACCACAGCTGGCATTCAAGGGT**AAAATTAATTGCTTCAGATACATGTGCTTCTTCTCAAAGTGCTGGACTTATTTGCATTGAGGAACATTCAGCAGAAGTAATCGATGCACACACAAAATGCGGCCACCCAATGA**CCCTGGCCTCCATCTCAAAGTCCCTTCCTCGTCCCCAAAGCCCTGACGGCTCAGCCATGCATGGAGGAACAAACACAGGTAGCGAGAGCAAATCAAACTGCAACAAAACACAGAAACATGAATGGGAAGCATCACAATGATGGGCTGGACGCAGCATAATGGGCCCGACAT

# HLXB9_1

TGTAGTCGGAGCGCTGTTTCAATAAACTCAAATTAATTTTTAAAATGAAAAAACATCATTTTTTTATCACCTAAATTGGGCTTAAAAATCTCCGTTGAAAAAAAAGGATTGCAAATCTGCCCAAACGCGTTTATGGGCTAAAAATAG**CGTAGGTCGCTTTAAATTGTGTTTTCATACCTTTTCCGGAAGGTAATTAATATTTAAACTATTCCGATTTGTCACTTTGATTTGTATTTTAGATAATTGTGAATGTAATTACGGAGCAAATTAACAGCTTTCAGGAAAGACCGGGTTCTGGGGTCCGGGTCCATCTGAGGCGCCTTATTAATTTTCTCTCCAATAGCTGTGATGAAAAGGGGCGATAAATCTGGGTGATCGGTGAAGGCCAATACTAAATGGCTCCATATTTCACCGCTGCTTTAATAGAAATATTCATGCGGGACACCTTAATGAAATTAAA**CCGGCGGAAGGCGCAGCTCGAACGGGACTCCGGCTCCGTGTCATCATCATGCGGGAGGCCGGTAACAAGCGCGTCGACGGCGCGTCTGCAAAACAAGACGTTTCTGGTAAATGTCCAGAGGTGAAAGTGTTTATTGAGCCCCCCACAACAACAGCACCGAGCCAAGGGACGCGGCGAATCAACGTATTTGTCAGTACGGCGCATTAACAGTTGCTATAAATACATTCTTGTGAAGGTAATGTTCATTTAAGACAAGGGCGAGAGCCCCCCGCGTCACGTTGACGCGTC

# HLXB9_2

TTCTCCCACGTTTCCATGCAGGTCCATGTCCCACCAGCATCAATCTCCCTCTTATCATTCTGTAATTGGTCATGATCATCAAGGGCCACGATTAAAGAGCGATCCTTTACGTGGAAACTACCCGC**TGACTTGTTGATTTCCCCCGAGCAAATAAACGTCCCTGGAGCGCGCTTAGTTGATGAATCGACAAAAACTAATCAGCTTTATTGGTAGACAGGTTAAGGGCAACTGGGTGTCAATAATTCTCATTTTGACCTCCTCTTCCATTAACT**TTAAGTGGCTTATTAGACCGAAGTCACCCGGAGCCTCCAACCTATTAGTTTGATCTCTCTCCTCATCACAGGTTTGGGGCTTTCGTGTGCACTTTTTTG

# HLXB9_3

ACATTATCCAACACTCGGTCGTAAACTGTGACTTAATGCATGCTTCATTTAAAAAAAAAAAACTGAAACATTTCCCTTGTTCAAGCCTCATTTGTGATGTTCTTTAGCTGCAAATATGCCACGTTTATTTTTCTATCGTGTATTTATAAAATGGGCATATACCAGTGCATGCTAACTTCTTGTTTAGCGTCGATCTCGGTGCAAAACCGTGTGAAATAAAATGTTTTTTTATATATAAAGTCACGAAGGCGCAGGCGTCTGTGATTTGTGGCCCTCTCGGCTGCAGAGGTCGGTGTTTTCCTGCGCGCTCTGACAGGTCGGGTCATG

KIAA0010_1

GATCAATCTCGGCCAATCGCCGCCGGGCAACAAGAGTCCGCAATAACGGCAAGTGCAAAATGGTGTAAGAGGCGCGTGGCGATGGGTTTGGAGCAGAACTACCCCCTGACTCC**AGGAATGAATAAGCATTTCTTAATTAGCCCATCGGGTTAAGTGCAAACCTTAATGGGCCTTTCCCAAACTCCGGCGTGACATCGTTCTCCAAACATGGCCAAGAAGAAAGTTCCATTAGCAATATTAATACAAGCTTAGTTGATCATTTTCATTCAATTAACTTCTTTCAATTAAATAGATAGCTTGACAATGGAGGTGATAAAAGATGGGGAGGGGTGGCTGATGACACGGACCAAACATTAGCGTGTTAGGGGTCAGTGGCGTTAAACTGACTGTAAATCCCACTGTTCAATTTGGAAAATGCATTTCCTCTGCTCTCCACTTCTTTAATGGGTCGTTTTAGCTGAC**AGGAGGCCTTTGAGCAGATCCTTTATGAAAACCCCCCGATACATTCTCAACATATTTGCCACATTGGCCAGAAAACTCTTGAATGGAAATGAAACACGAAAAACAACCAAGGAACATAACGGCAAGCTTCGTATTCTGGAT

KIAA0010_2

ACAGCAGCGGTGATGCAATCTCAATCTATATAAACGCTTGCCTTTTATCCGGGCGATAACGGCACTAAACAAATATCAATACGCCTCTCAAATGGCCACTCAAACGTCCATTTTTATGGAGAAAGAGGGACTTAAGGAAGGGTTAATTGAAAGTCAAATTCAAATTAATTTTCCCCGCCTAATCGCCTGAGCCATTAGCTATTGAGAGCACGATATCGAGGGGGCGGTCAGTTTGTAGCTGCTGATTGATCACTTGAAGAGGAGGTAGAAGTACAGAACCAGGAGGAGGAGGAGGAGGAGGAGGATGTTCTGATATGTGGAACAGAGCATCTCTGGCCAAGCAAGAGAGAAGAGGCCTGGATATCTGA**CTGTAAAACAGATCAAAATGCTATTACCACATGCTTTGAATGCTTATTTATGTCCTGTCGGCGAGGGCTGGCTGATTTAGTGCCAGCCAAATGCAACCGCTACATAAACAAGAGGCAGGAATCATCGGCTAATGGAAGCCCTAATTGAGAGGCAACTTCTTGTCATGAGGTAATTCTCCTCGGGGCTGAGATAGCCCCTCGCTGGGAAGGCATCCTGGTAAACAATCA**GCAGCACGTGCATTAAGGATGATTTAAGGACATTAGGCTCTAATAAATAGTGCAATCACAGGAAGGGAGGGACGGAAAAACAATGCCGTCAACGGATTATGCACAGGGTTAATTAACGAAACTGGAGCTGGGCTGGAGTTACAACCCCAACCCCCGCAGACAGATTCATAAACTAATGATGGCAATTCCTCTGAACAATGTCAGCCT

KIAA0010_3

TCATCCAGGTGACTCATGAGCCCCAAAGCTAATGTACTGGAAGAGTCCAAAAAGGAGCCCATTGGAGGCACTAA**AATATACAGGTCCTTATAAATGATAGCACTAAAAACCCTGACCGCATCAGCTAAATGGATTATAAATCCAGAGGATTTCCATAATGCATGCATCTTAAACACACAGGAGCGCATCAATCACAGCTATTTTCTAAACACGGGTTAATTGCGCAACTTCAAACATTGTCTATTCTTTATGCAAAATAATCTACTTA**ATGTTTGCGACATGGTGGGTGCTCATTTGGGACCATATGTGCCGGCCAGCCCCTTGAAACCTATCAATAACACTTCTGCAGGGAAGAGGCTGCAAAAAAAAACCTGTAGGCTTATTTTTTCACTGCCACTTACTTTA**TTTATGGATGTTTATTGCCAGAAATTGTGCCAAAATGACTTCGGCTGGAAAGAATTAATTACCGCGGCTCGGGCCTTTTAATATAAGTGAGTGCAGTGGCAACGCAATTAAAGCAGATCGCAGCACCATCAGGTGGAATAAATACCGGCCACGTTGCAGGTGCGTTCTGTTAGCTGCCAAATACATGGCTTAATTTACAACATTAAGAATCAATAAAATTATAATGCCTTTCAAACGTTTCTCTATCAATC**AGGGCCCACCTTGTGCACTTCCGAAGGGAATTATCAGAGACCAATGAGAGGAGAAGAGGAAGGGGAGGAGGAGGAGGAGGAGGAGCTAAAAGTGTAGAGACGCAGCGTCTGAAGAGGTTGGAGGTTTTGTACTAATGAGAGATGAATGAGGCTCAAAGAGAACCTGGCGTGGGCTTTCTTAC

SHH_1

ACCATACACACAAACAGTCCAAACAACCTTTTTTTATGGGCCCGATGAAAAGTTTCCTTTATTTCTAACACACGGCTCGCGGTTGTGTGACTTTTTAAAATGCTTTGCTGCCTTGATTGACTTTGATGTATTCAGTCAGAACCGGCCACAACAGATGGCCGAGGAGAGGAGGGCTAACGGCGAGACCTGTCAATCCGGAGAGTCCTCTAAGAAAACAAAGTGAAGACCCGGCTCTGGGAGAGGAGGAAACCCCTGAGCTCCTCAAGAGGTTGCGGCATTCTTGAGATTAGGGCAAGAAAAAC**GCAGACCACTGCTGTTAATGCTCCAGACAAAGGCGTGGAACAATTTATTAGTTAAGAATTCAAGCTCAGGCCTCAATCAAAGGCCTGTCGTCCACAATGGCAGACTTGGAGCTTTGGTTTGTTTTTTTAAAGTAAATAATCACGCTTAAAAGATACGGCAAAGATGGGGCTCAAGTCCCCCATAAAGCCCACATTCTCAACTGTCATCAGAATGGATTTCAGCGTGCGCTTTTATTTTCAACAAGTGCATTGTTTGCCTTTTTCTTTATGAATACCGTTTTAAAGAGCTTTTTATTTAACTTTCTTCCCCCTGAACCTCTTTACCTGATCTGTGGTATTTTCTGAAAAGACAGCTCACCTTGACGTGCAA**

SHH_2

AGCCATCATTACAGTGGAACTCCTAATGATTAGACATGTCCTTTGTAAATGAAATACAAATCACCTCAGGCTTTTGGGTTTA**CTTCATTTACTAAGTGCTACATTACCTCATACTTTGGACCTAACAATAGATTTCCCATAAATCTCCTGGTAGTTGAAATGATGAAGGGACTCACATATCAAGAAATGTGCCAGTGCTTATTAGCCCTTGAGAAGTTATTGAT**CGA

SHH_4

ATGTTTGAGATCAATGCCAGATG**CTCAAGACTGTTTTGATTTCCCACTTCATGGAGAACGTGAGCGTAATCTCCTCAGGATTCCC**CCTCCAGAGGTCTTTAGCTGCCATAAGCCACCATTCAACAGGCAAAACCCAACATGGGCTTTATACAGCCTGATGGTATTAATGGATGA

SHH_6

AGTGACTCCTCCATGTGTCCCCTTTCAAAGAATGAATGGAG**CCTTAAAAAAAAAAGTTTCTCTCTGAATAATTTATTCTCATGTGAACTCGCTGCTGCCGCGCAAATGGACTCTGAAACGGCGCGAGCAGCAAACTGTGCATAATCTATTTTTGTATCTCACAGACACAGAGAAAAGAGACGGAGGGGGAAAAAAAAGACAAAATCCTAAAAAAACATGAAAAACACACCGCTGAGAGAGGAGGAGATGTAGAGAGGAGCTACCTGACAGGCTGTAATGTTCATATGTGCATACATGTGCAACTGACTGTTATATTTCGGGGGAGAACGAACATGGGGTTCCATAAATTATATTTTTATACACAGAATTGTAAATTAGATTTTGACAGATCGCTACCGAATCACATTTCGTTATTTGAAATAGTGTAAGATATGAGAATATATTTTAATTTAA**ATACAGTAGCTGGGAAAGTATTGGAAAAAAAAAAATCCATCTTGTACTGCTTTGGTTTGATTCAGAATCGAATTCTTTCTGGAGACTGTTGGTTCCAGGTTGTCGGAGAGGGACAGATATTCTGCCTCACGTCTGCATTCTCT

CONTROLS

>SOX21 non-coding_1

TCATGACCGAGTCCGAGTGCAGGTTAGCATCAAAACCATTAAAATGAGAGTCCTGGTTGA

GTCAGAGCCATGACAAGTGTTTATTCATGTGCTCCGAATCATTGCTCATCAAAAACAAAG

TCTTTTGTTTCGGGCTTTTGTTTAGGAGTAGAAAACTGCACTTTGCTAAATATTTTAAAA

CCTGTCATTTATTTTTGAATGATTTACAATGACAACTTAGACCATCATAGGAAAAGTGAG

TAATAGATTGTCTTCAGAATCAGCAATTATTGCAGGCGTCTGTTGACATTAAAGGCTGAC

ACAGTCTATTCTACTATTTTGGAAGTTGGATGTGTCTGACATTCAAATGCTCTTGTTTTT

ATTCACACTTTGAGCCTTGTATGTATATTTCCTCACCGCTCTGACCTGCACCAGTC

PAX6 non-coding_1

CGCAGCGAGTCACCCGAGCGCCTCTAAAAAAGTTGTTGTTGCGGTGGGGACGTGCGTGCGTGCGAGCCACAAAAATGCTTTCGAACATCTATTCCTAAGAAGATGAAATAACTTATACATTTACGGCAATTGTGTAATATTTTAAAAATAATCCTGCAGGTTTACAGTCACGTTTAAAATACTTTTAATATCATCAAATTTGATCTTTCCGTCGCTCGATCCGGAACTCGAGTTTTCTACAAATTAAAGGAATAAATACACACATTAATTTGTAGAAATATTAACATTTAAATCAATAAATTGAATTCTACAGTTCAGATAATTCTCTCCGAATATATTAATAATTAGGATTGGAAAATGGCATTTTAAAATGAACATCATTTGTCAACAATTATGGTGGCTGTTTAGGAATCAGAACGAACCTGTTGACTTGGTGCGTGATGATGATTAATTCAATATCCTGTAAATGTTCAGCTATTTCTGACAACGGCCGACGCACAGTGAG

>PAX6 non-coding_2

GCTGAAACGTCATCGGAGCCAGTGGGGCGCACATTTGATGAGTGGACATCGGTCTTCAGA

TTTGTTTTAACCTATATAAGTGGTGGTTGTTCAGAAGGGAACCAAATGTAAAGTGCAATA

AAGTGCTGCACACTCACCGGATGAATGTCAGTTTGGGTATCCTGCTCGCGTAAAGCCGCT

TTCTCCTTCCGCGGGTCTTTCCCTCCGCGCGTCAGCGACACAAAATTATAGGTCCCGATT

AAAAATCTATCTGCGTCGGATCCGTCGTGGAGTTGAAATGTCAATGAGAGTGAGGGAAAA

AAATGCGCTTCTCTTACCAGTTACTTTCCCTTCAGCATCAGCCTCAAGTTGGCAGTGATT

GGCTGCAGAGACGCGGCCACGTCACACCTTACAGGACACGGACGCGCCGAACTGGGAAGT

GTGCAAGGTGCATGTGGCGCTCACCGGCCTGTTTATTGTCCGCTGGCGCGCGTGCACGTC

GTGTATACGTGCGTGTACCGATGAGAGAATTTACTTTTAGATTTTTTGTAATTTTCTAGG

TTAGTTCATTGAAGTAAAATATGGTTTGTGTGTGTGTGTGTGTGTGTGTGCGTGCGTGTG

TGTGTGTGTGTGTGTGTGTGTGTGTGCTGATATTATTTGTATAATTATTATTATGTAGTT

TCCCGTTGTGTCATTAATCATTTTATTTCTAAGGTGTTATTGTTAAATTATTTAATAGTC

GTAGTAGAATTCGTATTTTACAAAATATTACAAATAAGTCTTAAGTATCCCATTGGTGCA

GGTGCTTTGTTTCATTTGAACAAATAAGGTTAAAATATTTTCTTATTTTTCTGTTGTTTT

TTTTTACAGTTTATTTATTATCAGGCTTCACCTTGATGACAGCAGTTGCTACAAACTGTG

ACTTTTCGATATTTATTCATAAAAAGTGGCGGTCGTGTTTTCCGTGAACCTGCAACAGGT

GTTGTGCAGGTAAACCTTGTGACTTATCTGAACCTCCAGCAGCGGGTTGGGGTCAGATGT

GCAGCTACATCCCGCCGTGGTTGTGTTTCGAGGCTGAACCACCATAAACATAAATGATGT

TATAAATAATGCCACTGTTTTAGCTAAAATCCCCTTTTTATGTCTACAGCCCTATAAAGT

TGCTATCCGGACTCGTAATGGAGCTTCAGTTTCCCTTTTACCAAGGCCGGCTAAAAGCCT

CAAATGCTGTTTCACGACCTCTGATGTTTGTGTCAATTTGAACAAGACGGTGAGTAAATG

GGCAA

>SOX21 coding_1

TCATCGATGAAGCCAAGAGGCTGCGAGCGATGCACATGAAGGAGCATCCAGACTACAAAT

ACAGACCAAGGCGGAAGCCCAAAACTCTGATGAAGAAAGATAAGTTCTCTTTCCCGGTGC

CCTACAATCTCGGAGAGCACGACGCGCTCAAAGTTAGCGGACTTTCCGTCGGAGGACTTT

CTGAGTCTGTCCTGGGGAACCCCGACAAGGCGGCGGCGGCCGCAGCGGCTGCGGCGGCCA

GGGTTTTCTTCAACCCGTCCATGTCCACGAACCCCTATTCATTTTTCGACCTGGGATCCA

AGATGACTGAGCTTGGCCCGCCTTCATTTCCATACGCGTCTCCGCTGGGCTACCCGCCGG

CGGGCGCCACGGCTTTCACGGGGACTGGCAGCGTCGGCGGCGGGACGCACACCCACACTC

ACTCACACCCGTCCCCGGGAAACCCAGGCTACATGATCCCATGTAAC

>PAX6 coding_1

ctgaatggttggctgcccatctgcatgaggaaaaagtcatttcatgtggcaaatcaaatc

atgcacataacttccagtcaatacacttacactggggatgttgtcgttggtgcagatgcc

ctccgacagaagtctgtcacggatctcccacgcaaaaatagatgggcactcccgcttgta

ctgcgcgattttcgcgaccacctctggtgtggcgaccctgggcttgctgccgcctattgc

tcgtggtcgtatagagccagtctcataatatctacccaagatcttgctcacacagccgtt

ggacacctggatgg

SHH coding_1

CTCCGTGATGAATCAGTGGCCCGGGGTGAAGCTGCGGGTCACGGAGGGCTGGGACGAGGA

CGGCCACCACTTCGAGGAGTCGCTGCACTACGAGGGCCGGGCCGTGGACATCACCACCTC

GGACCGGGATAAGAGCAAATACGGCACCCTGTCCAGGCTGGCGGTGGAGGCCGGCTTCGA

CTGGGTCTATTATGAGTCCAAAGCTCACATCCACTGCTCCG
