# Supplementary material for: Highly Conserved Non-Coding Sequences Are Associated with Vertebrate Development
Source: PLoS Biol. 2004 Nov 11;3(1):e7. doi: 10.1371/journal.pbio.0030007 (PMC526512; doi:10.1371/journal.pbio.0030007)
Supplement: Table S2 — (67 KB DOC). [file pbio.0030007.st002.doc]

**Table S2**

**Statistically over-represented GO terms for genes located closest to the CNEs**

| GO category | GO ID | GO term | Genes next to CNEs^*^ | All human genes^+^ | P-value |
| --- | --- | --- | --- | --- | --- |
|  |  |  |  |  |  |
| Total Number with GO terms | | | 242 | 24020 |  |
|  |  |  |  |  |  |
| Molecular function | 0003700 | transcription factor activity | 85 | 1229 | 0 |
| Biological process | 0006355 | regulation of transcription, DNA dependent | 116 | 2802 | <10^-65^ |
| Biological process | 0006351 | transcription, DNA dependent | 116 | 2880 | <10^-63^ |
| Biological process | 0045499 | regulation of transcription | 116 | 2900 | <10^-62^ |
| Biological process | 0019219 | regulation of nucleobase, nucleoside, nucleotide and nucleic acid metabolism | 116 | 2926 | <10^-61^ |
| Biological process | 0006350 | transcription | 116 | 3045 | <10^-58^ |
| Biological process | 0019222 | regulation of metabolism | 116 | 3065 | <10^-57^ |
| Biological process | 0006366 | transcription from Pol II promotor | 27 | 459 | <10^-10^ |
| Biological process | 0006357 | regulation of transcription from Pol II promotor | 19 | 243 | <10^-9^ |
| Biological process | 0007399 | neurogenesis | 23 | 419 | <10^-8^ |
| Molecular function | 0003712 | transcription cofactor activity | 15 | 242 | <10^-5^ |
| Molecular function | 0003714 | transcription corepressor activity | 9 | 84 | <10^-5^ |
| Biological process | 0007417 | central nervous system development | 8 | 111 | <10^-3^ |

^*^ Number of genes with this GO term for genes closest to each CNE

^+^ Number of genes with this GO term for all human genes
